# Artificial Bacteriophages for Treating Oral Infectious Disease via Localized Bacterial Capture and Enhanced Catalytic Sterilization

**DOI:** 10.1002/advs.202400394

**Published:** 2024-08-19

**Authors:** Xiaocan Liu, Danfeng Luo, Shuang Dai, Yanting Cai, Tianyan Chen, Xingfu Bao, Min Hu, Zhen Liu

**Affiliations:** ^1^ Jilin Provincial Key Laboratory of Tooth Development and Bone Remodeling School and Hospital of Stomatology Jilin University Changchun 130021 China; ^2^ Beijing Advanced Innovation Center for Soft Matter Science and Engineering College of Life Science and Technology Beijing University of Chemical Technology Beijing 100029 China; ^3^ Key Laboratory of Pathobiology Ministry of Education Jilin University Changchun 130021 China

**Keywords:** artificial bacteriophage, bacterial inhibition, Fenton‐like catalytic reaction, oral infectious disease, virus‐like nanomaterials

## Abstract

With the rapid emergence of antibiotic‐resistant pathogens, nanomaterial‐assisted catalytic sterilization has been well developed to combat pathogenic bacteria by elevating the level of reactive oxygen species including hydroxyl radical (·OH). Although promising, the ultra‐short lifetime and limited diffusion distance of ·OH severely limit their practical antibacterial usage. Herein, the rational design and preparation of novel virus‐like copper silicate hollow spheres (CSHSs) are reported, as well as their applications as robust artificial bacteriophages for localized bacterial capture and enhanced catalytic sterilization in the treatment of oral infectious diseases. During the whole process of capture and killing, CSHSs can efficiently capture bacteria via shortening the distance between bacteria and CSHSs, produce massive ·OH around bacteria, and further iinducing the admirable effect of bacterial inhibition. By using mucosal infection and periodontitis as typical oral infectious diseases, it is easily found that the bacterial populations around lesions in animals after antibacterial treatment fall sharply, as well as the well‐developed nanosystem can decrease the inflammatory reaction and promote the hard or soft tissue repair. Together, the high Fenton‐like catalytic activity, strong bacterial affinity, excellent antibacterial activity, and overall safety of the nanoplatform promise its great therapeutic potential for further catalytic bacterial disinfection.

## Graphical abstract

Novel virus‐like copper silicate hollow spheres are well developed as efficient artificial bacteriophages for localized bacterial capture and enhanced catalytic sterilization in the treatment of oral infectious diseases. During the whole process of capture and killing, the artificial bacteriophages can efficiently capture bacteria, produce massive ·OH around bacteria, and induce an admirable antibacterial effect.

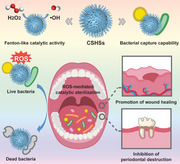

## 1 Introduction

Bacterial infections have been regarded as one of the biggest threats to human health, and they can cause ≈1 million deaths every year.^[^ ^1^ ^]^ For instance, over 10% of the adults in the United States have suffered from oral mucosal infection and periodontitis, which can lead to the disorders of immune, nervous, and cardiovascular systems.^[^ ^2^ ^]^ Since the discovery of penicillin in the 1920s, various newly developed antibiotics have extremely decreased the mortalities induced by bacterial infections. However, the abuse of antibiotics has also induced severe multidrug resistance and disturbance of microbial balance for different bacterial strains, narrowing their therapeutic windows.^[^ ^3^ ^]^ Therefore, the development of innovative bactericides that can overcome multidrug resistance and maintain the microflora balance is still in urgent demand.

With the rapid advances in nanotechnology, nanomaterials with Fenton or Fenton‐like catalytic activity have emerged as robust nano‐sized antibiotics against bacterial infections.^[^ ^4^ ^]^ These nanocatalysts usually hold great performance in chemodynamic sterilization owing to the catalytically generated hydroxyl radicals (·OH) in the presence of H_2_O_2_.^[^ ^5^ ^]^ Together with other therapeutic strategies, such as photothermal, photodynamic, sonodynamic, and microwave‐assisted approaches, several synergistic catalytic sterilizations have been well developed with ideal outcomes.^[^ ^6^ ^]^ Although promising, some drawbacks of above nanocatalyst‐based antibacterial platforms still remain and highly restrict their medical usages.^[^ ^7^ ^]^ First, ·OH generated from the nanocatalysts can only cause irreversible damage toward bacteria very close to it because of its ultra‐short lifetime and limited diffusion distance. Second, it is difficult to achieve satisfactory antibacterial effects due to the nonspecific targeting of most nanocatalysts and the presence of flowing liquid environments around wounds. Third, the release of foreign metal ions and organic components from nanocatalysts that do not exist in the body can cause potential toxicity. Against these battles, it is crucial to create efficient nanocatalysts with great bacterial targeting capability, high performance in catalytic sterilization, and safe chemical components.

Localized bacterial capture can extremely increase the number of nanomaterials around bacteria and improve the antibacterial efficacy of current nanosystem by killing pathogens in situ.^[^ ^8^ ^]^ For instance, borate‐contained nanoprobes can bind the bacterial peptidoglycan and capture bacteria.^[^ ^9^ ^]^ Antimicrobial polymers with positive charges can target the negatively charged pathogen membrane and selectively kill bacteria over mammalian cells.^[^ ^10^ ^]^ Furthermore, nanosystems modified with special functional groups, antibody, aptamer, and targeting peptide hold great affinity toward pathogens.^[^ ^11^ ^]^ However, above strategies mainly involve complicated synthesis and the additional use of biological reagents. More importantly, even though the distance between nanomaterials and bacteria is shortened, above approaches cannot well achieve the embedding of nanomaterials into the bacterial membrane that can furthest decrease the quenching effects of ·OH in the whole antibacterial process. Recently, viruses are widely utilized in the design of drug delivery systems.^[^ ^12^ ^]^ Apart from their nanoscale size and capsid protein, the shape and roughness of viruses are also considered as the determinants for their invasion capability. In general, virus‐like nanomaterials hold enhanced delivery performance, better affinity toward bio‐membranes, and higher barrier‐crossing ability.^[^ ^13^ ^]^ Taking inspirations from viruses, we envision that virus‐like nanocatalysts can achieve improved antibacterial performance by minimizing their distance from bacteria, decreasing the quenching effects of ·OH, and increasing the antibacterial efficacy.

**Scheme 1:**
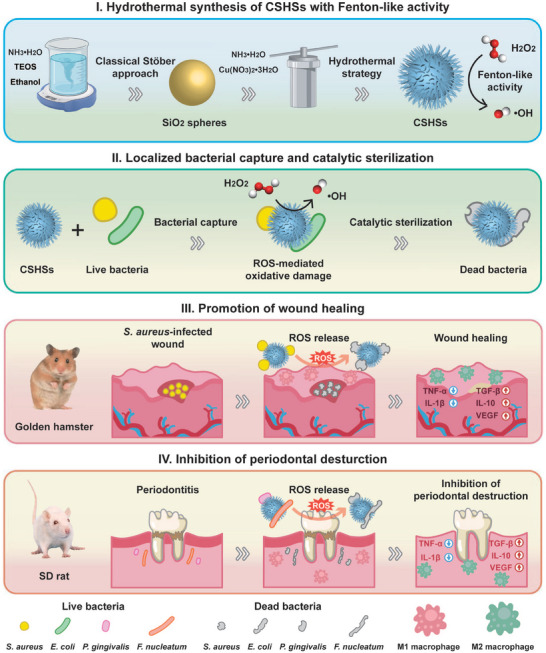
Schematic illustration for the synthesis of CSHSs with Fenton‐like catalytic activity, localized bacterial capture and catalytic sterilization, as well as their admirable biomedical usages in the promotion of *S. aureus*‐infected wound healing and the inhibition of periodontal destruction.

To prove our hypothesis, we created novel virus‐like copper silicate hollow spheres (CSHSs) and explored their usages as robust artificial bacteriophages for localized bacterial capture and enhanced catalytic sterilization in the treatment of oral infectious diseases (**Scheme** **1**). Based on the experimental result and theoretical calculation, these CSHSs could serve as ideal Fenton‐like nanocatalysts with broad‐spectrum antibacterial activity. Moreover, the virus‐like morphology of CSHSs endowed them with a high affinity toward bacteria and superior bacterial capture function. By using mucosal infection and periodontitis as typical oral infectious diseases, our nanosystem could efficiently eradicate pathogenic bacteria, alleviate inflammatory response, and promote tissue repair. To the best of our knowledge, this study was the first example that virus‐like nanomaterials could serve as artificial bacteriophages with high safety for enhanced catalytic sterilization in the field of stomatology. Thus, our findings presented an efficient approach toward the development of virus‐like nanocatalysts for the targeted treatment of oral infectious diseases and localized improvement of oral microenvironment.

## 2 Results and Discussion

### 2.1 Synthesis and Characterization of CSHSs

**Figure 1:**
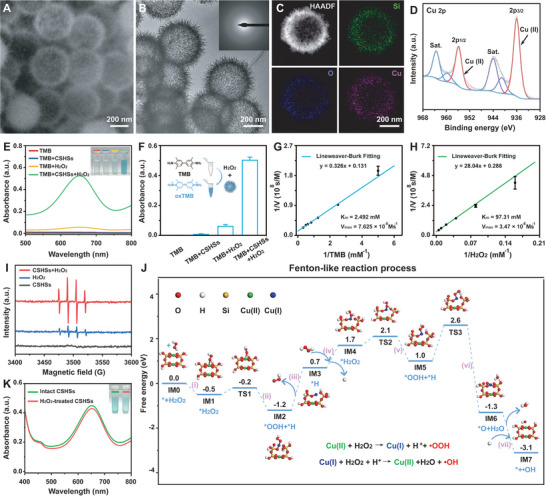
Characterization, Fenton‐like catalytic activity, catalytic mechanism, and stability of CSHSs. A) SEM image, B) TEM image, C) elemental mapping, and D) high‐resolution Cu 2p spectrum of CSHSs. Inset of (B), SEAD of CSHSs. E,F) UV–vis absorption spectra and relative absorbance values of TMB solutions after different treatments. G,H) Double‐reciprocal plots of activity of CSHSs at a fixed concentration of one substrate versus different concentrations of the second substrate for TMB or H_2_O_2_. I) ESR spectra for the ·OH detection from different systems. J) The schematic diagram for Fenton‐like catalytic mechanism of CSHSs+H_2_O_2_ system and relative Gibbs free energy diagrams. K) UV–vis absorption spectra of TMB solutions containing intact CSHSs or H_2_O_2_‐treated ones. Data in (F), (G), and (H) were presented as mean ± SD (*n* = 3).

Prior to the synthesis of CSHSs, colloidal silica spheres (SiO_2_ spheres) were first prepared using a classical Stöber method.^[^ ^14^ ^]^ Monodispersed SiO_2_ spheres held a spherical shape with an average size of 423 ± 19 nm and a uniform elemental composition of Si and O (Figure S1, Supporting Information). Then, CSHSs were synthesized via a facile hydrothermal strategy by using SiO_2_ spheres as a sacrificial template.^[^ ^15^ ^]^ Under a high temperature, ionization of ammonia could generate OH^−^, break Si─O bonds, and produce SiO_3_^2−^. After dissolving the outer layer of SiO_2_, copper silicate species formed gradually around the residual silica centers, resulting in the formation of CSHSs. Scanning electron microscopy (SEM) and transmission electron microscopy (TEM) images showed that CSHSs exhibited a virus‐like morphology with radially aligned nanospikes and a distinct hollow structure (**Figure** **1**A,B). By counting at least 100 particles, the average size of CSHSs was ≈440 nm (Figure S2A, Supporting Information). Energy dispersive spectroscopy (EDS) mapping and relative spectrum of CSHSs suggested the uniform distribution of Cu, Si, and O (Figure 1C; Figure S2B, Supporting Information). X‐ray photoelectron spectroscopy (XPS) spectra showed Cu 2p, Si 2p, and O 1s in CSHSs (Figure 1D, Figure S2C, Supporting Information). In the high‐resolution Cu 2p spectrum, two fitting peaks at 935.94 and 955.79 eV were ascribed to the 2p_3/2_ and 2p_1/2_ of Cu (II) with satellite peaks (Sat.), respectively. Significantly, we could detect the presence of Cu (I) after optimal peak fitting. These results therefore demonstrated that copper in CSHSS was all +2. Zeta potential indicated the negative charge of CSHSs (Figure S2D, Supporting Information). X‐ray diffraction (XRD) patterns revealed that CSHSs held the same diffraction peaks with standard hydrated copper silicate (JCPDS No. 03–0219) while SiO_2_ spheres had an amorphous structure (Figure S2E, Supporting Information). Above diffraction peaks of CSHSs were manifested by the selected area electron diffraction (SAED) result (Figure 1B, inset). Evidenced by the Fourier transform infrared (FT‐IR) spectra, peaks at 805 and 1100 cm^−1^ resulted from the symmetrical stretching vibration and asymmetric stretching vibration of Si─O in SiO_2_ spheres (Figure S2F, Supporting Information). Meanwhile, peaks at 807 and 1042 cm^−1^ were linked to the symmetrical stretching vibration and asymmetric stretching vibration of Si─O in CSHSs. These slight differences in FT‐IR spectra demonstrated the presence of strong interaction between copper ions and SiO_2_ during the synthesis of CSHSs. These exciting results thus indicated the successful synthesis of CSHSs.

### 2.2 Fenton‐Like Catalytic Activity of CSHSs

After understanding the properties of CSHSs, we explored their Fenton‐like catalytic activity and relative catalytic mechanism. 3,3 ´,5,5 ´‐tetramethylbenzidine (TMB) was utilized as typical chromogenic substrate to verify our hypothesis. CSHSs could catalyze the generation of ·OH in the presence of H_2_O_2_, and TMB was oxidized into blue oxTMB by ·OH (Figure 1E). Compared with H_2_O_2_ alone, ·OH could induce more color shift (Figure 1F). Because reaction conditions might affect the catalytic effect of Fenton‐like reactions, the optimal pH value and temperature were further explored. As shown in Figure S3 (Supporting Information), the Fenton‐like catalytic activity was highest at pH 4.0 and 50 °C. Notably, CSHSs might hold high catalytic activity in oral mildly acidic environment (pH = 6.6–7.1) and normal body temperature (37 °C). Moreover, the Fenton‐like catalytic activity of CSHCs followed a concentration‐dependent manner, whereas the absorbance at 652 nm was positively linked with the levels of various reagents (Figure S4, Supporting Information). Steady‐state catalytic kinetics parameters including the Michaelis–Menten constant (K_m_) and the maximum reaction velocity (V_max_) were evaluated at varied concentrations of H_2_O_2_ and TMB (Figure S5, Supporting Information). For H_2_O_2_ substrate, K_m_ and V_max_ could be calculated as 97.31 mm and 3.47 10^−8^ ms^−1^ while K_m_ and V_max_ were 2.492 mm and 7.625 10^−8^ Ms^−1^ for TMB substrate (Figure 1G,H; Table S1, Supporting Information). Significantly, the K_m_ value of CSHSs was much lower than that of classical Fe_3_O_4_‐based nanozymes with a K_m_ value of 154 mm, indicating the presence of a much stronger affinity between CSHSs and H_2_O_2_.^[^ ^16^ ^]^ Subsequently, 2,2 ´‐azino‐bis (3‐ethylbenzthiazoline‐6‐sulfonic acid) diammonium salt (ABTS) and o‐phenylenediamine (OPD) were selected as another two chromogenic substrates to re‐confirm above results. As expected, both ABTS and OPD could be catalytically oxidated into colored products with absorption peaks at 417 and 430 nm (Figure S6, Supporting Information). By using 5,5‐dimethyl‐1‐pyrroline N‐oxide (DMPO) as a capture reagent, the generation of ·OH was confirmed using electron spin resonance (ESR) analysis. Four characteristic signal peaks with an intensity ratio of 1:2:2:1 were detected in the samples of H_2_O_2_ and CSHSs+H_2_O_2_, which demonstrated the production of ·OH (Figure 1I). Compared with H_2_O_2_ alone, CSHSs could extremely increase the ·OH generation by decomposing H_2_O_2_ within the same incubation period. All these results indicated the favorable Fenton‐like catalytic efficiency of CSHSs.

Density functional theory (DFT) calculation was then employed to explore the potential mechanism responsible for the generation of ·OH. Typically, a cluster model was constructed and developed to simulate the unit containing Cu sites.^[^ ^17^ ^]^ Figure 1J provided the proposed mechanism of Fenton‐like catalytic process and relative Gibbs free energy profile. The initial state of the Fenton‐like reaction involved CSHSs and H_2_O_2_ was defined as intermediate 0 (IM0). i) H_2_O_2_ was adsorbed onto the Cu(II) sites of CSHSs. ii) After transition state 1 (TS1), the adsorbed H_2_O_2_ decomposed into hydroperoxyl (^*^OOH) on Cu(II) site and hydrogen (^*^H) on O site. Meanwhile, the Cu(II) species were reduced to Cu(I). iii) ^*^OOH dissociated from its binding site to generate ·OOH. iv) Above generated Cu(I) absorbed another H_2_O_2_, and ^*^H dissociated from its binding site to generate H^+^. v) After TS2, H_2_O_2_ dissolved into ^*^OOH and ^*^H on their relative binding sites. vi) After TS3, H_2_O was generated and dissociated from the system, resulting in the production of ^*^O. vii) Residual ^*^O adsorbed H^+^ in the medium and generated ·OH while the Cu(I) species were oxidized to Cu(II). In the whole catalytic process, the generation of ·OOH in the iii) step held the highest energy barrier of 1.9 eV, which was considered as the rate determining step (RDS). Moreover, there were two processes involved in the decomposition of H_2_O_2_ with energy barriers of 0.3 and 0.4 eV, indicating that above two processes were not the RDSs and easy to cross. Last but not least, chemical process was concluded in Figure 1J to benefit the understanding.

To verify their stability and catalytic activity, H_2_O_2_‐treated CSHSs were created at first. According to the spectroscopic analysis, H_2_O_2_‐treated CSHSs and CSHSs held similar catalytic activity (Figure 1K). Moreover, there were no differences in morphology and crystal structure between these two samples (Figure S7A, Supporting Information). XPS analysis indicated a slight increase of the Cu(I) amount after the H_2_O_2_ treatment, which further confirmed the presence of Cu (I)/Cu(II) cycle during the Fenton‐like catalytic process (Figure S7B,C, Supporting Information). In detail, four fitting peaks at 932.9, 935.8, 952.7, and 955.6 eV could be ascribed to the Cu (I) 2p3/2, Cu (II) 2p3/2, Cu (I) 2p1/2, and Cu (II) 2p1/2 while other peaks were satellite peaks of Cu(II). Thus, our well‐developed CSHSs could maintain their high stability and catalytic activity after the H_2_O_2_ treatment.

### 2.3 Toxicity and Biocompatibility Evaluation

Before the biomedical usages of CSHSs, we explored their biocompatibility and long‐term toxicity.^[^ ^18^ ^]^ CSHSs exhibited no obvious growth inhibition on L929 fibroblasts (L929 cells) and rat bone marrow mesenchymal stem cells (rBMSCs) even at a high dose of 80 µg mL^−1^ (Figure S8, Supporting Information). In the presence of CSHSs, all the hemolysis rates were lower than 5%, a standard non‐hemolytic percentage in the clinic (Figure S9A and Table S2, Supporting Information). As the key indices of coagulation function, prothrombin time (PT) and thrombin time (TT) were tested to describe the effect of CSHSs toward plasma. The addition of CSHSs did not show any effect on PT and TT (Figure S9B,C and Table S3, Supporting Information). To confirm their long‐term toxicity, CSHSs were administered intraperitoneally or intragingivally to mice. During the whole experimental period, all groups gained weight and no significant differences occurred among these groups (Figure S10, Supporting Information). Hematoxylin‐eosin (H&E) histologic examination implied that no organ damage was found among these groups (Figure S11, Supporting Information). Results of hematology, blood biochemistry, and urinalysis indicated no significant differences across above groups at the end of experiment (Figure S12 and Table S4, Supporting Information). Accordingly, CSHSs held overall biosafety in mice within the investigated concentration range.

### 2.4 Catalytic Antibacterial Effect and Bacterial Capture Function of CSHSs

**Figure 2:**
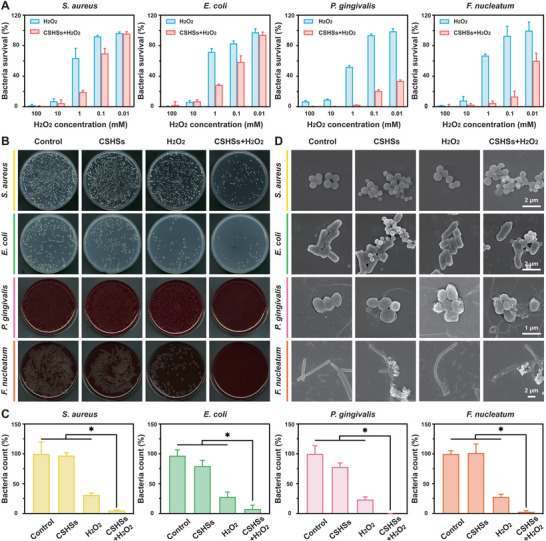
In vitro antibacterial activities. A) Concentration‐dependent survivals of *S. aureus*, *E. coli*, *P. gingivalis*, and *F. nucleatum* after different treatments. B,C) Photos of agar plate containing colonies formed by the bacteria collected from different groups and relative quantitative results based on bacteria count. D) SEM images of various bacteria after different treatments. Data in (A) and (C) were presented as mean ± SD (n = 3). Statistical significance was calculated using one‐way ANOVA with multiple comparison tests. ^*^ *p* < 0.05.

*Staphylococcus aureus* (*S. aureus*), *Escherichia coli* (*E. coli*), *Porphyromonas gingivalis* (*P. gingivalis*), and *Fusobacterium nucleatum* (*F. nucleatum*) were selected as typical bacteria to evaluate the in vitro antibacterial activity of our design. It was well known that H_2_O_2_ with a mass concentration of 3% was widely utilized as a lotion to treat mouth ulcers and other oral infectious diseases. Compared with wide‐spectrum antibiotics, H_2_O_2_ held inexpensive instinct and non‐drug resistance. Therefore, H_2_O_2_ in our design was considered not only as a control group, but also as a drug commonly utilized in the clinical practice. **Figure** **2**A revealed that low concentrations of H_2_O_2_ did not exhibit ideal antibacterial activity. However, the bacterial viabilities decreased sharply after the addition of CSHSs. Notably, H_2_O_2_ with a concentration of 0.1 mm held a better antibacterial activity in the presence of CSHSs compared with those of H_2_O_2_ alone. Results according to bacteria count implied the highest antibacterial activity of our CSHSs+H_2_O_2_ system while H_2_O_2_ alone only held a certain level of activity (Figure 2B,C). Meanwhile, the CSHSs group showed a similar antibacterial ability to the control group. Based on the images of live–dead staining, compared with the groups treated with CSHSs or H_2_O_2_ alone, our CSHSs+H_2_O_2_ system had the most red fluorescence spots, also indicating its best antibacterial activity (Figure S13, Supporting Information). SEM images revealed that the bacterial membrane became rough and wrinkled after the treatments with CSHSs or H_2_O_2_ alone (Figure 2D). However, our CSHSs+H_2_O_2_ system could severely destroy the integrity of the bacterial membrane and some bacteria seemed fully lysed. The above damages could be ascribed to the generated ·OH from our system, which was also confirmed using DCFH‐DA staining. Compared with other groups, both *S. aureus* and *E. coli* treated with CSHSs+H_2_O_2_ showed green fluorescence with high intensity (Figure S14, Supporting Information). These results implied that our CSHSs with high Fenton‐like catalytic activity could increase the antibacterial efficacy of H_2_O_2_.

**Figure 3:**
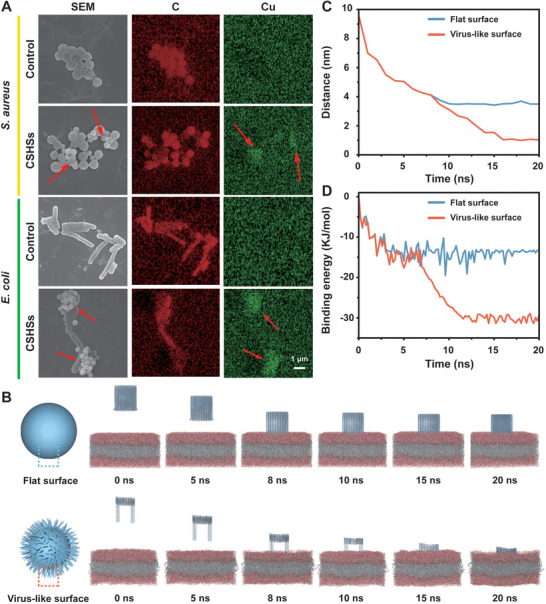
Bacterial capture performance and bacterial membrane penetration simulations. A) SEM images and relative elemental mapping patterns of different bacteria incubated with CSHSs. The presence of CSHSs was marked by red arrows. B) Representation for the bacterial membrane penetration processes of different spheres with a flat surface or a virus‐like surface. C,D) Time‐dependent changes of the mass distance and the binding energy between the bacterial membrane and two different surfaces.

After understanding the antibacterial activity and ·OH‐involved antibacterial mechanism of CSHSs+H_2_O_2_ system, its antibacterial activity was then explored in depth. Both SEM and fluorescent images showed that there was a strong interaction between CSHSs and bacteria (**Figure** **3**A; Figure S15, Supporting Information). In detail, the agglomerates with adhering CSHSs and bacteria could be easily found after treatment while bacteria were still well dispersed without the addition of CSHSs, further implying the outstanding bacterial capture capability of CSHSs. However, CSHSs and the above bacteria were all negatively charged, indicating that the strong interaction between CSHSs and bacteria was independent of their electrical interaction (Figure S16, Supporting Information). Earlier studies revealed that particles with special shape held different biological effects as compared with spherical ones. Moreover, mammalian cells and bacteria were more likely to adhere or attach to rough surfaces. Therefore, molecular dynamics (MD) simulations were utilized to examine the interaction between virus‐like CSHSs and bacteria. Owing to the complexity of real interactions, simplified models including bacterial membranes surrounded by H_2_O, spheres with flat surfaces (size: 5 × 5 nm), and spheres with virus‐like surfaces (spike diameter: 0.4 nm, spike length: 5 nm, space between neighboring spikes: 4.2 nm) were well developed (Figures S17 and S18, Supporting Information). Our simulation began with that two different spheres were placed at equal distances outside the membrane (Figure 3B). A pulling force was then utilized to enable the above two structures to move toward the membrane. Actually, the pulling force was ascribed to the collisions between bacteria and spheres caused by the Brownian movement, van der Waals force, and physical vibration during the mixing.^[^ ^19^ ^]^ As expected, a virus‐like surface could readily impale the membrane and damage it. In contrast, the flat surface could not be inserted into the membrane while the membrane still kept its integrality. The penetration depth of the above two structures crossing the membrane was quantified by monitoring the distance between the membrane core and the surface. Two surfaces could contact the exterior of membrane within 8 ns. After that, the flat surface's distance remained constant while the virus‐like surface continued to approach the interior of membrane. The distance curve of virus‐like surface did not reach its plateau level even after 15 ns while the distance curve of flat surface reached at 10 ns, demonstrating the better permeability of virus‐like surface against membrane (Figure 3C). This phenomenon was subsequently confirmed by evaluating the interaction energy between membrane and two surfaces. When virus‐like surface approached the membrane, the interaction energy gradually decreased (Figure 3D). At 20 ns, the interaction energy of virus‐like surface reached its lowest value of −30 kJ mol^−1^. However, the flat surface held a higher interaction energy of −13 kJ mol^−1^, implying the stronger interaction between membrane and virus‐like surface. These results indicated the high permeability into bacterial membrane of our virus‐like CSHSs, which not only benefited for shortening the ·OH diffusion distance but also enhanced the antibacterial effect.

### 2.5 In Vivo Antibacterial Therapeutic Performance in Oral Mucosal Infection Model

**Figure 4:**
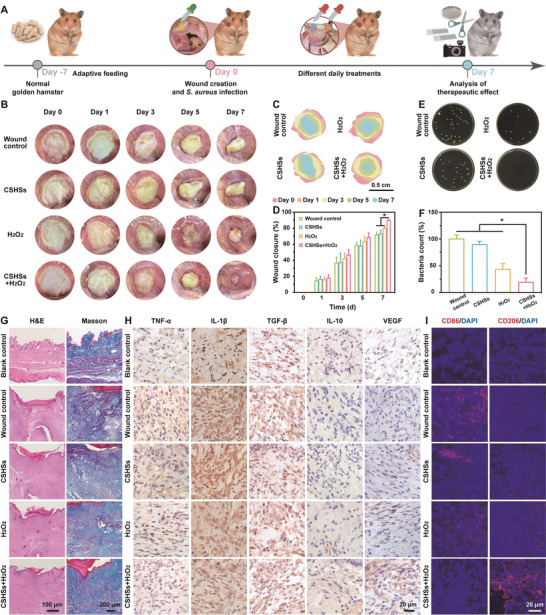
Establishment of rodent model with oral mucosal infection and relative evaluation of CSHSs+H_2_O_2_ system‐assisted antibacterial therapy. A) Schematic illustration of in vivo *S. aureus*‐assisted infection and different treatments in golden hamsters. B) Photos of wounds on days 0, 1, 3, 5, and 7 in golden hamsters receiving different treatments. C,D) Traces of wound closure over 7 days for different groups and the corresponding quantitative results. E,F) Photos of colonies formed by the bacteria collected from wounds after different treatments and the corresponding bacterial numbers calculated by colony counting. G) H&E and Masson staining images of wounds after different treatments. H,I) Immunohistochemistry and immunofluorescence images of wounds in different groups harvested at the experimental endpoint. Data in (D) and (F) were presented as mean ± SD (*n* = 3). Statistical significance was calculated using one‐way ANOVA with multiple comparison tests. ^*^ *p* < 0.05.

Because of the excellent antibacterial activity, our CSHSs+H_2_O_2_ system was then utilized to treat oral mucosal infection in golden hamsters. **Figure** **4**A showed the establishment of the animal model and relative therapeutic schedule. In order to eliminate the differences in individual oral microbiota and achieve a similar oral environment, golden hamsters adapted to diet for 7 days before the modeling. Both photos of agar plates and quantitative results of colony counting indicated that there were no differences in bacterial counting between various experimental groups, which thus could ensure the completion of the following experiments (Figure S19, Supporting Information). The successful establishment of mucosal infection model was provided in Figure S20 (Supporting Information). As a kind of common Gram‐positive pathogenic bacteria in oral cavity, *S. aureus* was selected and utilized to infect the wounds. Figure 4B,C indicated that our CSHSs+H_2_O_2_ system held the minimum wounds among all the groups during the whole therapeutic process. With time passing, the wounds treated by CSHSs+H_2_O_2_ could rapidly scab without swelling while other groups still released sanies. As expected, the scar sizes in the CSHSs+H_2_O_2_ group were the smallest, and corresponding wounds almost healed on the 7th d. Figure 4D suggested that the CSHSs+H_2_O_2_ group had the highest healing rate (90.11 ± 1.62%) as compared with the groups of wound control (72.15 ± 3.06%), CSHSs (73.51 ± 4.13%), and H_2_O_2_ (80.13 ± 1.83%). To assess the antibacterial efficacy of the above groups, fluid from oral mucosal lesions after treatment was collected for bacteria count. The CSHSs+H_2_O_2_ group had the smallest bacterial colonies and the difference was ≈5 times lower than that of the wound control group, which indicated that the admirable wound healing efficacy of our design could be ascribed to its outstanding antibacterial activity (Figure 4E,F). Furthermore, we recorded the time‐dependent counting of white blood cells (WBCs) and relative C‐reactive protein (CRP) content after the antibacterial treatment. Figure S21 (Supporting Information) showed that both the number of WBCs and the corresponding content of CRP increased sharply at the early stages of treatment while they gradually decreased along with the time passing. All these exciting results implied that our CSHSs+H_2_O_2_ nanosystem could reduce systemic inflammation through antibacterial therapy.

Subsequently, mucosal tissues receiving different treatments and a normal one were taken for histological and immunohistochemical analysis (Figure 4G,H). The normal mucosal tissue was collected from a healthy golden hamster without any treatment, which was defined as the group of blank control. Compared with the healthy mucosal, tissues in the groups of wound control, CSHSs, and H_2_O_2_ all emerged large areas of necrocytosis and fragmentary cells. However, the decrease of inflammatory cells and increase of fibroblast cells was noticed in the CSHSs+H_2_O_2_ group, indicating the formation of new epithelium. The new generation of collagen after the wound healing process was further explored via Masson staining.^[^ ^20^ ^]^ Similar with the wound control group, the groups of H_2_O_2_ or CSHSs revealed a relatively lower amount of collagen. In contrast, the CSHSs+H_2_O_2_ group held a significant collagen deposition with dense and ordered structures. Considering the importance of macrophage polarization in wound healing, the expression of pro‐inflammatory factors (TNF‐α and IL‐1β) and anti‐inflammatory factors (TGF‐β and IL‐10) in above samples were discussed.^[^ ^21^ ^]^ The decrease of pro‐inflammatory factors and the increase of anti‐inflammatory factors could be detected in the CSHSs+H_2_O_2_ group, suggesting the presence of tissue repair via the reduction of bacteria‐induced immune response (Figure S22, Supporting Information). Meanwhile, Figure 4I revealed that the CSHSs+H_2_O_2_ group held the lowest level of CD86 (M1 marker) and highest level of CD206 (M2 marker), further implying that the antibacterial treatment localized at wound sites could highly reverse the inflammatory microenvironment. Detailed quantitative information of CD86 and CD206 in oral mucosal infection model was provided in Figure S22 (Supporting Information). As an essential biomarker for angiogenesis in the tissue repair, the expression of vascular endothelial growth factor (VEGF) was also evaluated.^[^ ^22^ ^]^ Compared with the healthy mucosal, similar VEGF expression was found in the CSHSs+H_2_O_2_ group. It was well known that the detection of inflammatory factors and in vivo ROS around wounds at different time points could provide an essential explanation for the inflammatory clearance during the whole therapeutic process. As shown in Figure S23 (Supporting Information), the expression of TNF‐α in the group of CSHSs+H_2_O_2_ gradually decreased along with the time passing while the expression of TGF‐β increased, which indicated the step‐by‐step occurrence of inflammatory clearance and a rapid response toward bacterial infection. According to the results of dihydroethidium (DHE) staining, the CSHSs+H_2_O_2_ group with the lowest ROS content on the 7th day after the first antibacterial treatment held the best therapeutic effect among all the groups, which could extremely alleviate the oral mucosal infection (Figure S24, Supporting Information). The therapeutic efficacy of our design was then assessed via radar chart analysis using ulcerations, bacteria, inflammation, bleeding, as well as collagen destruction as typical indexes (Table S5, Supporting Information). As expected, the CSHSs+H_2_O_2_ group held the smallest area, also implying its best therapeutic efficacy (Figure S25, Supporting Information). Furthermore, the absence of histological changes based on the H&E staining of main organs indicated the overall safety of our system (Figure S26, Supporting Information). Therefore, the utilization of CSHSs+H_2_O_2_ system could efficiently eradicate bacteria and accelerate the healing of infected wounds.

It was well known that both Gram‐positive and Gram‐negative bacteria had the opportunity to infect the wounds when oral mucosa was damaged. After understanding the therapeutic effect of our CSHSs+H_2_O_2_ system on *S. aureus*‐assisted infection, its performance on Gram‐negative bacteria‐assisted infection was further evaluated in detail. As a kind of Gram‐negative bacteria in oral cavity, *E. coli* was introduced to establish a wound infection model after the adaptive feeding of golden hamsters (Figure S27, Supporting Information). Similar to its outcomes on *S. aureus*‐assisted infection, our well‐developed CSHSs+H_2_O_2_ system also held admirable antibacterial effects against *E.coli*. It could be easily found that our antibacterial system could extremely promote wound healing after the killing of pathogenic bacteria around the wounds. All these results thus indicated that the combination of CSHSs and H_2_O_2_ could result in considerable antibacterial outcomes in the treatment of oral infectious diseases induced by both Gram‐negative and Gram‐positive bacteria.

### 2.6 In Vivo Antibacterial Therapeutic Performance in Rat Periodontitis Model

**Figure 5:**
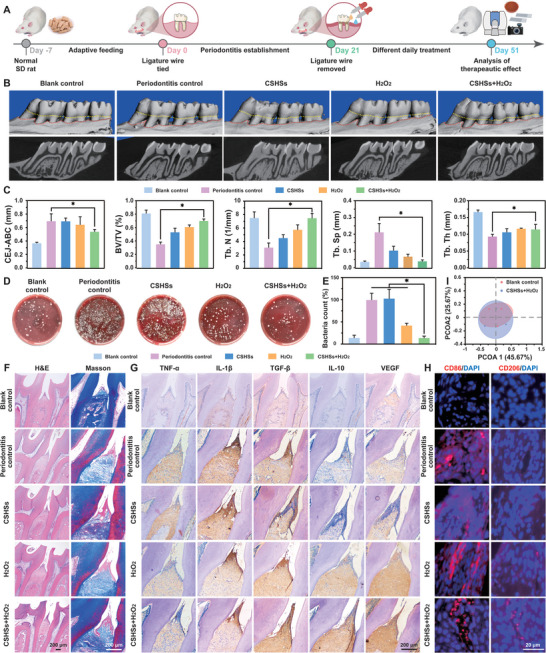
Establishment of rat periodontitis model and relative evaluation of CSHSs+H_2_O_2_ system‐assisted antibacterial therapy. A) Schematic illustration of the preparation of animal model and relative schedule for in vivo treatments. B) Micro‐CT images of collected maxilla blocks in different groups. C) Quantitative analysis of CEJ‐ABC, BV/TV, Tb. N, Tb. Sp, and Tb. Th in different groups. D,E) Photos of colonies formed by the bacteria collected from different groups and the corresponding bacterial number calculated by colony counting. F) H&E and Masson staining images of tissues collected from different groups. G,H) Immunohistochemistry and immunofluorescence images of tissues harvested from different groups. I) PCoA plot to visualize the β‐diversity of oral microbiome in different groups at the experimental endpoint. Data in (C) and (E) were presented as mean ± SD (*n* = 3). Statistical significance was calculated using Student's *t*‐test or one‐way ANOVA with multiple comparison tests. ^*^ *p* < 0.05.

Next, we explored the antibacterial efficacy of our CSHSs+H_2_O_2_ system in a rat periodontitis model. **Figure** **5**A illustrated the establishment of the animal model and relative therapeutic schedule. Normal rats without any treatment were defined as the blank control group. Because efficient antibacterial treatment could reduce alveolar bone damage and relieve soft tissue inflammation, the antibacterial effect of our system was then assessed via micro‐computed tomography (Micro‐CT) and histopathological analysis.^[^ ^23^ ^]^ A photo of rat periodontitis model was shown in Figure S28 (Supporting Information). As an essential index, the vertical distance between alveolar bone crest (ABC) and cementoenamel junction (CEJ) was tested and quantified after high‐resolution 3D reconstruction. The inflammatory control group had the longest distance between ABC and CEJ (0.70 ± 0.01 mm) among all the groups, indicating the severe destruction of alveolar bone (Figure 5B,C). Compared with the periodontal condition, the two groups of CSHSs and H_2_O_2_ showed negligible recovery in the height of alveolar bone. Notably, the CSHSs+H_2_O_2_ group held the most obvious reduction in alveolar bone destruction (0.54 ± 0.03 mm) owing to its remarkable antibacterial efficacy. Other bone parameters including bone volume per tissue volume (BV/TV), trabecular number (Tb. N), trabecular separation (Tb. Sp), and trabecular thickness (Tb. Th) were analyzed to re‐confirm our above results.^[^ ^24^ ^]^ In contrast with the inflammatory control group, the CSHSs+H_2_O_2_ group could maximize the values of BV/TV, Tb. Th, and Tb. N, and decrease the value of Tb. Sp, indicating that our design could highly reduce bone loss after treatment. Moreover, fluid from the gingival sulcus was collected after daily disinfection and subjected to bacteria count. Figure 5D,E demonstrated that the number of colonies in the CSHSs+H_2_O_2_ group was less than 15% of that in the inflammatory control group.

According to the H&E and Masson‐stained images of periodontal tissues, obvious collagen loss and lots of inflammatory cells could be found in the inflammatory control group (Figure 5F). Meanwhile, our CSHSs+H_2_O_2_ system could greatly improve the anomalies and enhance the collagen deposition, which was highly consistent with the above bone parameters. Results of immunohistochemistry staining additionally demonstrated that the expressions of TNF‐α and IL‐1β were the lowest in the CSHSs+H_2_O_2_ group while the expressions of TGF‐β, IL‐10, and VEGF were the highest among the experimental groups except for the blank control group, which implied the satisfactory anti‐inflammatory effects of our design (Figure 5G; Figure S29, Supporting Information). Meanwhile, the CSHSs+H_2_O_2_ group held the lowest level of CD86 and the highest level of CD206, indicating that the antibacterial treatment could reverse the inflammatory microenvironment in rat periodontitis model. Moreover, runt‐related transcription factor 2 (Runx2) and osteocalcin (OCN) staining was then utilized to confirm the immuneregulation of our design on the bone regeneration in the rat periodontitis model. Compared with the other experimental groups except for the blank control group, the CSHSs+H_2_O_2_ group held a higher expression of Runx2 and OCN at the regions with periodontitis modeling, which indicated that its admirable antibacterial treatment could augment the repair of hard tissue (Figure 5H; Figure S30, Supporting Information). By using bone loss, bacteria, inflammation, collagen destruction, and periodontal condition as indexes, radar chart analysis indicated the smallest area of the CSHSs+H_2_O_2_ group, re‐confirming its best antibacterial efficacy in periodontitis model (Figure S31 and Table S6, Supporting Information). H&E staining of main organs from above groups promised the safety of the current therapeutic strategy (Figure S32, Supporting Information). To assess the impacts of our design on oral microbiota, the abundance, diversity, and colony structure in the groups of blank control and CSHSs+H_2_O_2_ were explored. Concluded by Simpson and Shannon indexes, no differences could be found in α‐diversity (Figure S33, Supporting Information). Meanwhile, β‐diversity represented by the principal coordinate analysis (PCoA) also suggested two similar clusters with negligible variations (Figure 5I). More importantly, no differences in the distribution of oral microbial communities were found at the phylum and genus levels (Figure S34, Supporting Information). Together, our system exhibited enviable therapeutic outcomes in rat periodontitis model by killing harmful pathogens, alleviating tissue inflammation, accelerating alveolar bone regeneration, and remodeling periodontal microenvironment.

## 3 Conclusion

In summary, we developed and employed novel virus‐like CSHSs as artificial bacteriophages for localized bacterial capture and enhanced catalytic sterilization. Confirmed by the chemical kinetics measurement and ESR spectra, these well‐prepared CSHSs could efficiently catalyze the generation of ·OH in the presence of H_2_O_2_. Evidenced by the experimental exploration and theoretical calculation, our CSHSs with abundant nanospikes had an admirable localized bacterial capture function. After shortening the distance between bacteria and CSHSs, the generation of massive ·OH around the bacteria endowed current system with excellent antibacterial performance. Notably, CSHSs together with trace H_2_O_2_ could achieve over 90% bacterial inhibition by damaging the integrity of bacteria. With the help of animal models with oral infectious diseases, our in vivo disinfection results further identified that CSHSs with ideal Fenton‐like catalytic activity could efficiently eliminate bacteria, reduce inflammatory reaction, and accelerate tissue repair. Overall, this study not only provided a deep insight into the localized bacterial capture function in the catalytic sterilization but also gave a new direction for the design of artificial bacteriophages.

## Conflict of Interest

The authors declare no conflict of interest.

## Supporting information

Supporting Information

## Data Availability

The data that support the findings of this study are available from the corresponding author upon reasonable request.
