# Supplementary material for: Artificial Bacteriophages for Treating Oral Infectious Disease via Localized Bacterial Capture and Enhanced Catalytic Sterilization
Source: Adv Sci (Weinh). 2024 Aug 19;11(41):2400394. doi: 10.1002/advs.202400394 (PMC11538703; doi:10.1002/advs.202400394)
Supplement: Supplementary file 1 — Supporting Information [file ADVS-11-2400394-s001.pdf]

ADVANCED SCIENCE
Open Access

Supporting Information

for *Adv. Sci.*, DOI 10.1002/advs.202400394

Artificial Bacteriophages for Treating Oral Infectious Disease via Localized Bacterial Capture and Enhanced Catalytic Sterilization

Xiaocan Liu, Danfeng Luo, Shuang Dai, Yanting Cai, Tianyan Chen, Xingfu Bao, Min Hu* and Zhen Liu**

Supporting Information

Artificial Bacteriophages for Treating Oral Infectious Disease via Localized Bacterial Capture and Enhanced Catalytic Sterilization

Xiaocan Liu, Danfeng Luo, Shuang Dai, Yanting Cai, Tianyan Chen, Xingfu Bao, Min Hu,* and Zhen Liu**

I. Experimental Section

Chemicals and materials. Copper (II) nitrate trihydrate ($Cu(NO_3)_2{\cdot}3H_2O$), ammonium hydroxide ($NH_3{\cdot}H_2O$), 3,3′,5,5′-tetramethylbenzidine (TMB), 5,5-dimethyl-1-pyrroline N-oxide (DMPO), 2,2′-azino-bis (3-ethylbenzthiazoline-6-sulfonic acid) diammonium salt (ABTS), o-phenylenediamine (OPD), Rhodamine PEG Thiol (RB-PEG-SH), 4',6-diamidino-2-phenylindoleand (DAPI), and ethylene diamine tetraacetic acid (EDTA) were purchased from Aladdin Reagent (Shanghai, China). Tetraethyl orthosilicate (TEOS), Calcein AM, propidium iodide (PI), and 2′,7′,-dichlorodihydrofluorescein diacetate (DCFH-DA) were obtained from Sigma-Aldrich (Shanghai, China). Fetal bovine serum (FBS) and Dulbecco's modified Eagle's medium (DMEM) were obtained from ACRO Biosystems (Beijing, China). The primary antibodies of CD86 antibody, TNF-α antibody, IL-1β antibody, IL-10 antibody, and TGF-β antibody were obtained from Abmart (Shanghai, China). The primary antibodies of CD206 antibody, Runx2 antibody, and OCN antibody were purchased from Proteintech (Wuhan, China). Alexa Fluor-conjugated secondary antibody and dihydroethidium (DHE) were purchased from Beyotime (Shanghai, China). Hamster hypersensitivity C-reactive protein (CRP) ELISA kit was purchased from YLKBIO (Shanghai, China). All chemicals were of analytical grade and utilized without further purification. Deionized water throughout all experiments was obtained *via* a Milli-Q water system.

Measurements. Field emission scanning electron microscope (SEM) instrument (Apreo 2C) equipped with an energy dispersive spectrometer (ULTIM Max65) was utilized to collect SEM images. Transmission electron microscope (TEM) instrument (Talos F200S) with an energy dispersive spectrometer (SUPER X) was utilized to read out the morphological information and relative mapping imaging at an accelerating voltage of 200 kV. Fourier transform infrared (FT-IR) spectrometer (NICOLET iS50) was utilized to record the FT-IR spectra of various samples. Wide-angle X-ray diffraction (XRD) patterns of different samples were collected on an X-ray

diffractometer (Rigaku Ultima IV). X-ray photoelectron spectrometer (ESCALAB 250Xi) was utilized to record the elemental composition of the samples. Absorption values or spectra of various samples were determined on an Ultraviolet-visible (UV-vis) spectrometer (Shimadzu UV-1800). Electron spin resonance (ESR) spectrometer (Bruker E-500 ESR) was utilized to explore the generation of radicals. Micro-computed tomography (micro-CT) analysis was performed on a PerkinElmer Quantum FX micro-CT.

Synthesis of SiO_2 spheres and CSHSs. Prior to the synthesis of copper silicate hollow spheres (CSHSs), colloidal silica spheres (SiO_2 spheres) were firstly prepared according to the classic Stöber method with some modification. For the formation of SiO_2 spheres with an average diameter of 380 nm, TEOS (5 mL), $NH_3 \cdot H_2O$ (10 mL), ethanol (65 mL), and deionized water (25 mL) was mixed in a flask. Above mixtures were then kept at room temperature under mild stirring for 6 h for the formation of SiO_2 spheres. After centrifugation, decanting of supernatant, and freeze-drying, the resulting SiO_2 spheres were collected for further use and characterization. Subsequently, CSHSs were synthesized using a hydrothermal strategy by using SiO_2 spheres as self-sacrificial templates. Typically, SiO_2 spheres (100 mg), $Cu(NO_3)_2 \cdot 3H_2O$ (0.75 mmol), and $NH_3 \cdot H_2O$ (1 mL) were dispersed in deionized water (50 mL) under stirring. Above suspension was transferred into a Teflon-lined stainless-steel autoclave and heated at 140 °C. After an overnight reaction, stainless-steel autoclave was cooled to room temperature naturally. The resulting CSHSs were rinsed with deionized water and ethanol in sequence, as well as freeze-dried overnight for further use.

Fenton-like activity and catalytic kinetics assay of CSHSs. The Fenton-like activity of CSHSs was verified with the help of various chromogenic reactions in the presence of H_2O_2 by using TMB, ABTS, or OPD as chromogenic substrates. Typically, different chromogenic substrates (1 mM) were mixed in acidic PBS (20 mM, 3 mL) containing CSHSs (100 μg/mL) and H_2O_2 (10 mM) at room temperature. After 1 h of co-incubation, color changes of above

mixtures were perceived by naked eye or monitored on a UV-vis spectroscopy. Absorbance values at 652 nm (TMB), 417 nm (ABTS), or 430 nm (OPD) were recorded, respectively. In addition, other experiments were performed to explore the Fenton-like activity of CSHSs at different pH values and temperatures, as well as their concentration-dependent manners. ESR spectrometer was utilized to confirm the production of short-lived ·OH by using DMPO as capture reagent. To further verify the steady-state catalytic kinetics of CSHSs, Michaelis-Menten constant (K_m) and the maximum velocity (V_{max}) were investigated according to the Lineweaver-Burk plots for H_2O_2 and TMB, respectively. Michaelis-Menten constant was well calculated by using the Lineweaver-Burk double reciprocal as the following formulas.

$$V = V_{max} \frac{[S]}{K_m + [S]}$$

$$\frac{1}{v} = \frac{K_m}{V_{max}[S]} + \frac{1}{V_{max}}$$

Stability of CSHSs. CSHSs (100 μg/mL) was co-incubated with H_2O_2 (10 mM) overnight, and then washed with ethanol and dried in vacuum. After above treatments, H_2O_2-treated CSHSs were submitted to a series of measurements including TEM, XRD, and XPS. Moreover, their Fenton-like activity was explored using TMB as chromogenic substrate.

Density functional theory (DFT) calculations. The DFT calculations were implemented using DMol3 program. The generalized gradient approximation (GGA) with a Perdew-Burke-Ernzerh of method (PBE) was utilized as the exchange-correlation functional.[1] The Effective Core Potentials (ECP) were used in the calculations for Cu atoms, whereas H, O and Si were treated as in the all-electron.[2] The double numerical plus polarization (DNP) was set as the basis set. The following convergence criterion was set: 1×10^{-5} Ha for total energy, 0.002 Ha/Å for force, as well as 0.005 Å for displacement. We assumed the Fenton-like catalytic mechanism and Shenzhen HUASUAN Technology participated in the whole calculation process.

Animals. Institute of Cancer Research (ICR) mice (female, 6-week-old), Sprague-Dawley (SD)

rats (female, 6-week-old), and golden hamsters (female, 8-week-old) were purchased from Jilin University Laboratory Animal Center (Changchun, China). All animal experimental procedures were approved by the Institutional Animal Care and Utilization Committee of Jilin University.

Cell culture. L929 fibroblasts (L929 cells) were purchased from Chinese Academy of Medical Sciences. Rat bone marrow mesenchymal stem cells (rBMSCs) were acheived in lab from the tibia and femur bone marrow of SD rats. Both L929 cells and rBMSCs were cultured in DMEM containing 10% FBS in a humidified incubator at 37 °C with 5% CO_2.

Cytotoxicity. Cell Counting Kit-8 (CCK-8) assay was utilized to quantify the cytotoxicity of CSHSs. Briefly, L929 cells or rBMSCs were seeded in a 96 well-plate with a density of 1×10^4 cells per well. After the overnight culture, medium was replaced with fresh DMEM containing 10% FBS and different concentrations of CSHSs. Following 24 h of incubation, cell viability was evaluated by CCK-8 assay and calculated as the following formula.

$$Cell\ viability\ (\%\ of\ control) = \frac{OD_{sample} - OD_{blank}}{OD_{control} - OD_{blank}} \times 100$$

Visible cytotoxicity. Live-dead staining was utilized for the directed observation of cellular viability of L929 cells in the presence of CSHSs. L929 cells were seeded in a 6 well-plate with a density of 1×10^6 cells per well at first. After the overnight culture, medium was replaced with fresh DMEM containing 10% FBS and different concentrations of CSHSs. Calcein AM and PI were utilized to stained L929 cells after different treatments, and fluorescence images were captured on a fluorescence microscope. Moreover, rBMSCs were utilized to evaluate the visible cytotoxicity of CSHSs and re-confirmed their in vitro biosafety.

Hemocompatibility. Hemolysis assay and coagulation assay were selected and performed to explore the hemocompatibility of CSHSs. For the hemolysis experiment, heparin-stabilized fresh blood was collected from healthy SD rats, and centrifuged to produce the red blood cells (RBCs). Subsequently, RBCs were diluted with 0.9% NaCl and different concentrations of

CSHSs were added into RBC suspension. After incubation at 37 °C for 3 h, all samples were centrifuged separately. Hemoglobin absorption at 541 nm of each sample was recorded, and the hemolysis rate was calculated as the following formula.

$$Hemolysis\ rate\ (\%\ of\ control) = \frac{OD_{sample} - OD_{saline}}{OD_{water} - OD_{saline}} \times 100$$

For the coagulation assay, prothrombin time (PT) and thrombin time (TT) of mixed plasma containing different concentrations of CSHSs were determined on an automated coagulation analyzer. Plasma from the same source was considered as the negative control.

Long-term toxicity. To explore the long-term toxicity of CSHSs, ICR mice were randomly divided into 3 groups including control (no treatment), intragingival instillation (100 μg/mL, 20 μL), and intraperitoneal administration (100 μg/mL, 1 mL). After above treatments, body weight and relative behavior of mice were carefully recorded. 30 days later, whole blood was collected from above groups for hematological analysis and serum biochemical analysis while urine was collected for urinary routine analysis. Then, mice were sacrificed and major organs were collected for further histological examination.

Antibacterial efficiency. *Staphylococcus aureus* (*S. aureus*, ATCC 29213), *Escherichia coli* (*E. coli*, ATCC 25922), *Fusobacterium nucleatum* (*F. nucleatum*, ATCC 25586), and *Porphyromonas gingivalis* (*P. gingivalis*, ATCC 33277) were utilized to evaluate the in vitro antibacterial ability of CSHSs in the presence or absence of H_2O_2. Typically, bacteria were cultured in the liquid brain-heart infusion (BHI) media, then collected, washed with 0.9% NaCl, and diluted to 1×10^6 CFU/mL. Subsequently, above bacteria were transferred into acidic PBS containing various concentrations of H_2O_2 with or without CSHSs (40 μg/mL). 10 min later, bacteria in above groups were carefully rinsed with 0.9% NaCl, re-suspended in fresh BHI liquid medium, and grown overnight at 37 °C. The concentrations of bacteria were estimated by measuring the optical density at 600 nm (OD_{600}) of bacterial suspensions. Relative bacterial

viability was calculated according to the following formula.

$$Bacterial\ viability\ (\%) = \frac{OD_{sample} - OD_{BHI}}{OD_{control} - OD_{BHI}} \times 100$$

Moreover, bacteria collected from BHI medium were randomly divided into 4 groups including control (bacteria without any treatments), CSHSs (bacteria treated with CSHSs), H_2O_2 (bacteria treated with H_2O_2), and CSHSs+H_2O_2 (bacteria treated with both CSHSs and H_2O_2). The concentrations of H_2O_2 and CSHSs in above experiments were 1 mM and 40 μg/mL, respectively. Receiving above treatments, a certain amount of bacterial suspension was plated on solid LB or Columbia blood agar plates, and the bacterial survival rates from these 4 groups were carefully monitored based on the results of colony counting.

Live-dead staining of bacteria. Bacteria collected from above 4 groups including control, CSHSs, H_2O_2, and CSHSs+H_2O_2 were stained with SYTO-9 and PI for 30 min at 37 °C in dark. After repeated washing with 0.9% NaCl, fluorescence images of live and dead bacteria were recorded with the help of a fluorescence microscopy.

ROS detection and morphological observation. DCFH-DA was utilized to verify the ROS in bacteria after different treatments. In detail, bacteria collected from above 4 groups were treated with DCFH-DA for 30 min in dark and washed with 0.9% NaCl. Subsequently, fluorescence microscopy was utilized to define the presence of ROS in bacteria. To detect the morphological changes of bacteria after different treatments, above samples were fixed with glutaraldehyde, dehydrated gradually by sequential concentrations of ethanol, and layered onto a silicon pellet for SEM analysis.

Bacterial capture experiment. Prior to the exploration of the bacterial capture capability of CSHSs, fluorescent CSHSs were prepared by adding RB-PEG-SH (1 mg) into the dispersion containing CSHSs (10mg/mL, 10 mL) for 6 h at first. Subsequently, fluorescent CSHSs were rinsed with deionized water, separated by centrifugation, and freeze-dried in dark overnight for

further use. After co-incubation with fluorescent CSHSs, bacteria were stained with SYTO-9 and observed under a fluorescence microscope. Meanwhile, samples containing the composites of bacteria and CSHSs were layered onto silicon pellets for the following SEM analysis.

Molecular dynamics (MD) simulation. The atomistic MD simulation was performed using the GROMACS 2022.5 package. In a simulation box of 17×17×30 nm^3, the bacterial lipid membrane was constructed using 1,2-dihexadecanoyl-rac-glycero-3-phosphocholine (DPPC). Flat surface and virus-like surface were simulated by copper silicate molecules to study the interactions and penetration process. In detail, the size of smooth plane was 5 nm×5 nm while the virus-like surface had a plane of 5 nm×5 nm with two spikes (spike diameter: 0.4 nm, spike length: 5 nm, space between neighbouring spikes: 4.2 nm). $GAFF_2$ force field was utilized to bring the surfaces toward the lipid bilayer at the rate of 0.02 nm/s.[3] Periodic boundary conditions in all directions were employed. The temperature was maintained at 298 K using the velocity rescale thermostat while the atmospheric pressure was set at 1.0 atm. The penetration process and the corresponding distance and binding energy data were recorded. We designed the MD simulation process and the Shenzhen HUASUAN Technology participated in the whole calculation process.

***S. aureus*-assisted oral mucosal wound model.** Oral mucosal infection model and relative treatments were well developed on golden hamsters. To extremely eliminate the differences in individual oral microbiota and achieve a similar oral environment before animal modeling, golden hamsters from the same purchase source adapted to diet for 7 days. Circular wounds with an average diameter of 7.5 mm were created in the right cheek pouch of golden hamsters after the adaptive feeding. *S. aureus* suspension (1×10^8 CFU/mL, 20 μL) was added dropwise to infect above wounds. After the successful establishment of oral mucosal infection model, golden hamsters were randomly divided into 4 groups including wound control (wounds treated with 0.9% NaCl), CSHSs (wounds treated with CSHSs), H_2O_2 (wounds treated with H_2O_2), and

CSHSs+H_2O_2 (wounds treated with CSHSs and H_2O_2). The concentrations of H_2O_2 and CSHSs in above groups were 1 mM and 40 μg/mL, respectively, while the total volume of above reagent was 100 μL. Moreover, normal golden hamsters without any treatment were defined as the group of blank control. Prior to the localized treatment of above wounds, concentrated solution containing H_2O_2 or CSHSs were prepared in 0.9% NaCl. Subsequently, formulations with different components were developed by rapid mixing above concentrated solutions as the requirement of different experimental groups. After that, mixtures with different formulations were administered onto the wounds drop by drop and the wounds were exposed to above medications at least 10 min. Daily treatments were given to above groups everyday and photos were taken at the same time. Wound closure rate was analyzed using Image J software and calculated according to the following formula.

$$Wound\ closure\ rate\ (\%\ of\ initial\ state) = \frac{Area_{day\ 0} - Area_{day\ n}}{Area_{day\ 0}} \times 100$$

Once after daily treatments on the 7th day, sterile cotton swabs were utilized to take bacteria from above 4 groups, and real-time antibacterial activity was recorded using the method of CFU counts. After the whole therapeutic period, all the golden hamsters were sacrificed and the samples from the above 4 groups were dissected for H&E staining, Masson staining, immunohistochemistry staining, and immunofluorescence staining. Meanwhile, sample from normal golden hamster in the group of blank control was collected to process histological analysis. Main organs from golden hamsters were collected for photos and H&E staining as a part of biosafety assessment of our therapeutic system.

In order to provide a clearer description for the clearance of infection and inflammatory, the in vivo ROS and the expression of inflammatory factors localized around the mucosal injuries at different time points were well investigated. According to our above design, similar grouping of golden hamsters and the corresponding therapeutic approach were performed. At the end of

each time point, DHE staining was utilized to detecte the in vivo ROS while TNF-α and TGF-β were selected as the typical inflammatory factors to describe the conditions of inflammatory clearance along with the antibacterial treatments. In addition, blood samples collected from the golden hamsters from the above groups at different time points were utilized for other tests including hematology and hypersensitivity C-reactive protein.

Immunofluorescence staining. Samples of cheek pouches were sliced and trimmed into serial sections. The slides were deparaffinized in xylene and rehydrated through ascending ethanol series. Subsequently, the sections were subjected to antigen retrieval using citrate buffer at 95°C for 20 min. To quench the endogenous peroxidase, the sections were treated with 3% H_2O_2 in methanol. After washing with Tris buffer, slides were permeabilized and blocked with goat serum at 37°C. For immunofluorescence staining, the above tissue sections were well incubated with primary antibody overnight at 4°C, followed by the addition of Alexa Fluor-conjugated secondary antibody. Finally, nuclei were stained with DAPI and the corresponding images were captured with the assistance of a fluorescence microscope.

***E.coli*-assisted oral mucosal wound model.** *E.coli*-assisted oral mucosal wound model was established to re-confirm the antibacterial efficacy of our well-developed CSHSs+H_2O_2 system. Experimental details and corresponding anti-infection therapy were performed according to a similar strategy utilized in the treatment of *S. aureus*-assisted oral mucosal wound model.

Periodontitis model. Ligature-induced periodontitis model was well established by tying orthodontic ligature wires sub-gingivally around the right maxillary first molars of SD rats. After 3 weeks feeding with glucose water, ligature wires were removed, and Micro-CT was utilized to evidence the successful establishment of rat periodontitis model. Subsequently, rats were randomly divided into 4 groups including periodontitis control (treated with 0.9% NaCl), CSHSs (treated with CSHSs), H_2O_2 (treated with H_2O_2), and CSHSs+H_2O_2 (treated with CSHSs and H_2O_2). The concentrations of H_2O_2 and CSHSs in above groups were 1 mM and 40

μg/mL while the total volume of above reagent was 100 μL. Meanwhile, healthy rats without any treatment were defined as the blank control group. Detailed daily treatment was similar with the strategy utilized in the *S. aureus*-assisted oral mucosal wound model. To further evaluate the real-time antibacterial efficacy of above groups, gingival sulcus fluid was obtained from the daily disinfected treatments and prepared for ager plate counting. After one month of daily treatment, all the rats were sacrificed according to the ethical requirements of experimental animals. Then, maxillas were collected and scanned using Microcomputed tomography (Micro-CT). Different indicators of periodontitis degree including the distance between the cementoenamel junction and the alveolar bone crest (CEJ-ABC), bone volume fraction (bone volume/tissue volume, BV/TV), trabecular number (Tb. N), trabecular thickness (Tb. Th), and trabecular separation (Tb. Sp) were measured carefully. Finally, all samples were fixed in 4% paraformaldehyde overnight, transferred to 10% EDTA for decalcification over a period of 3 months, as well as prepared for the following analysis including histological staining, immunohistochemistry staining, and immunofluorescence staining. Meanwhile, main organs of rats were collected for photos and H&E staining as a part of biosafety assessment of our therapeutic system. Maxilla sample from normal rat was collected as the blank control.

Microbiota profiling. At the end of the whole experiment, oral samples were collected from the gingival sulcus and 16S rRNA gene amplification was prepared for the following oral microbiological analysis. RNA sequencing was performed using Illumina MiSeq platform (Sangon Biotech Co., Ltd., Shanghai, China). After the extraction of microbial DNA, the V3 to V4 regions of bacterial 16s RNA gene were amplified using polymerase chain reaction. Paired-end 2×250 bp fragments were sequenced, and operational taxonomic units with 97% similarity were clustered using a Bayesian algorithm. Evaluation metrics for α diversity were utilized as post hoc tests to verify the significance of differences while principal coordinate analysis (PCoA) for β diversity was utilized to determine the differences between different samples.

Statistical analysis. Statistical analysis was carried out using OriginPro 2022 and GraphPad Prism 8. Significant differences were analyzed using the Student's *t*-test for two groups or one-way analysis of variance (ANOVA) for multiple group comparisons. All data in this study were expressed as mean ± standard deviation (SD) from at least 3 independent experiments. The *p* value less than 0.05 was considered statistically significant. The "ns" meaned no significant difference.

References.

[1] J. Perdew, K. Burke, and M. Ernzerhof. Generalized gradient approximation made simple. Physical Review Letters, 1996, 77 (18): 3865.

[2] M. Dolg, U. Wedig, H. Stoll, and H. Preuss. Energy-adjusted *ab initio* pseudopotentials for the first row transition elements. The Journal of Chemical Physics, 1987, 86 (2): 866.

[3] D. Vassetti, M. Pagliai, and P. Procacci. Assessment of GAFF2 and OPLS-AA general force fields in combination with the water models TIP3P, SPCE, and OPC3 for the solvation free energy of druglike organic molecules. Journal of Chemical Theory and Computation, 2019, 15 (3): 1983.

II. Supporting Figures and Tables

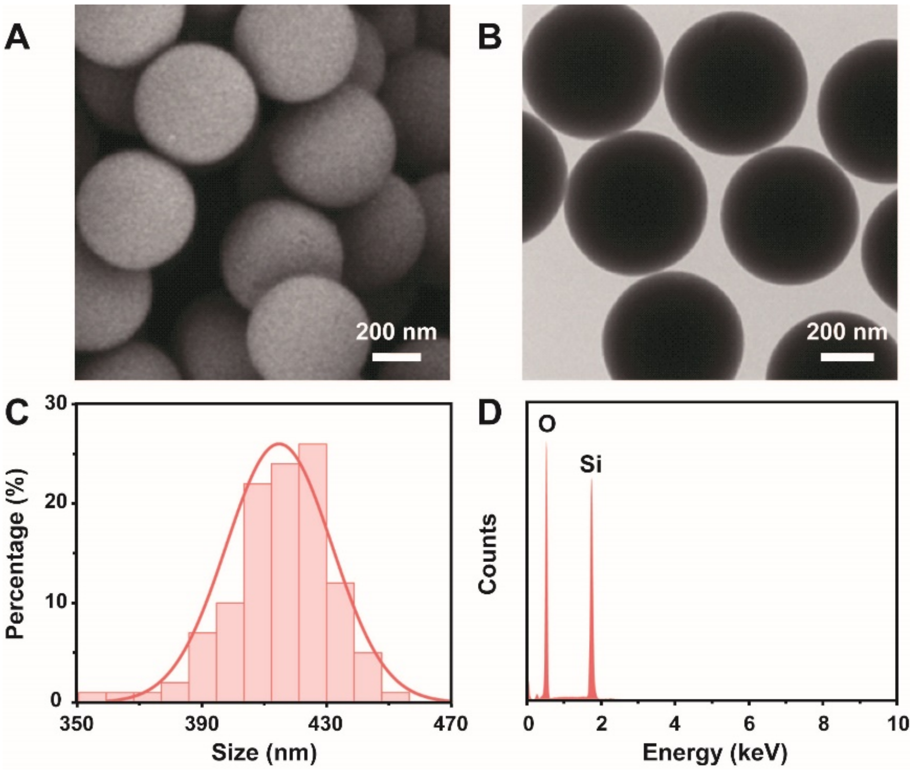

Figure S1. SEM image (A), TEM image (B), size-distribution (C), and EDS spectrum (D) of SiO_2 spheres.

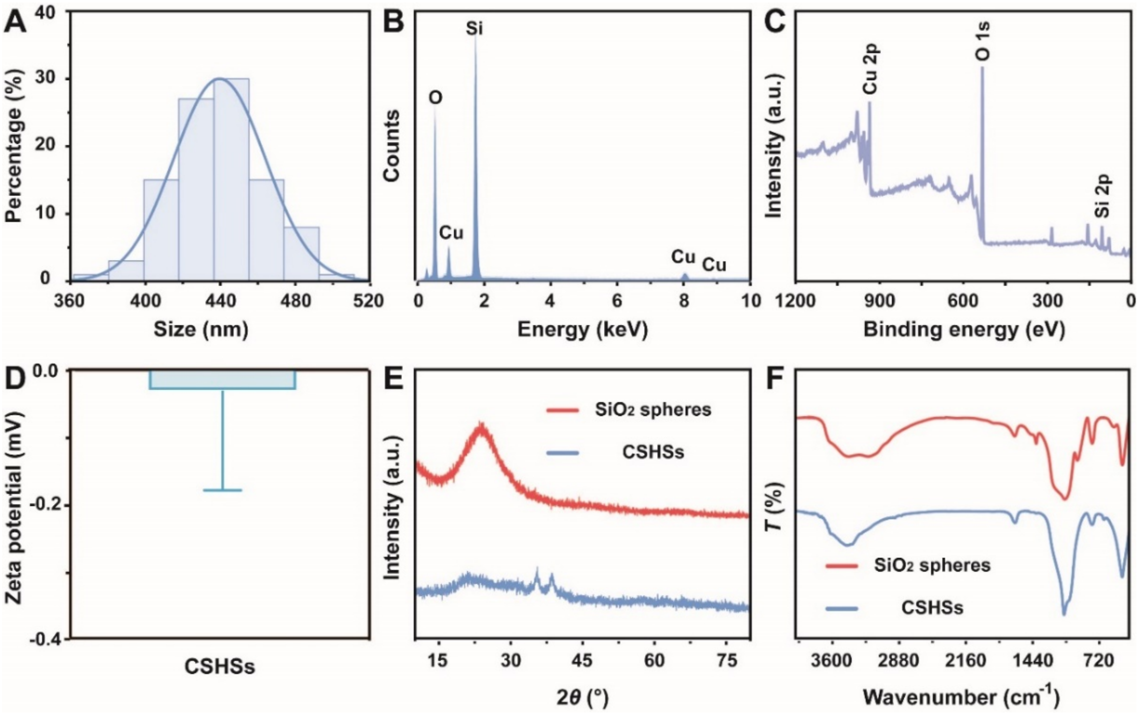

Figure S2. Size-distribution (A), EDS spectrum (B), XPS spectrum (C), and zeta potential (D) of CSHSs. XRD patterns (E) and FT-IR spectra (F) of SiO_2 spheres and CSHSs. Data in (D) were presented as mean ± SD (n = 3).

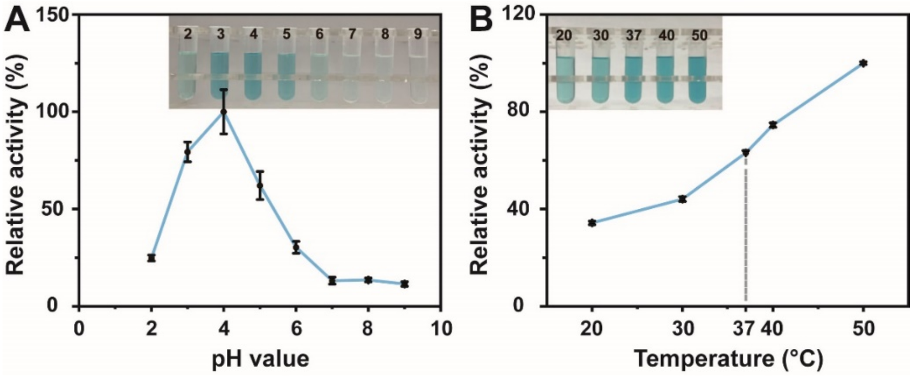

Figure S3. The pH value-dependent (A) and temperature-dependent (B) catalytic activity of CSHSs+H_2O_2 system. The concentrations of CSHSs, TMB, and H_2O_2 utilized in the typical experiment were 100 µg/mL, 1 mM, and 10 mM, respectively. Data were presented as mean ± SD (n = 3).

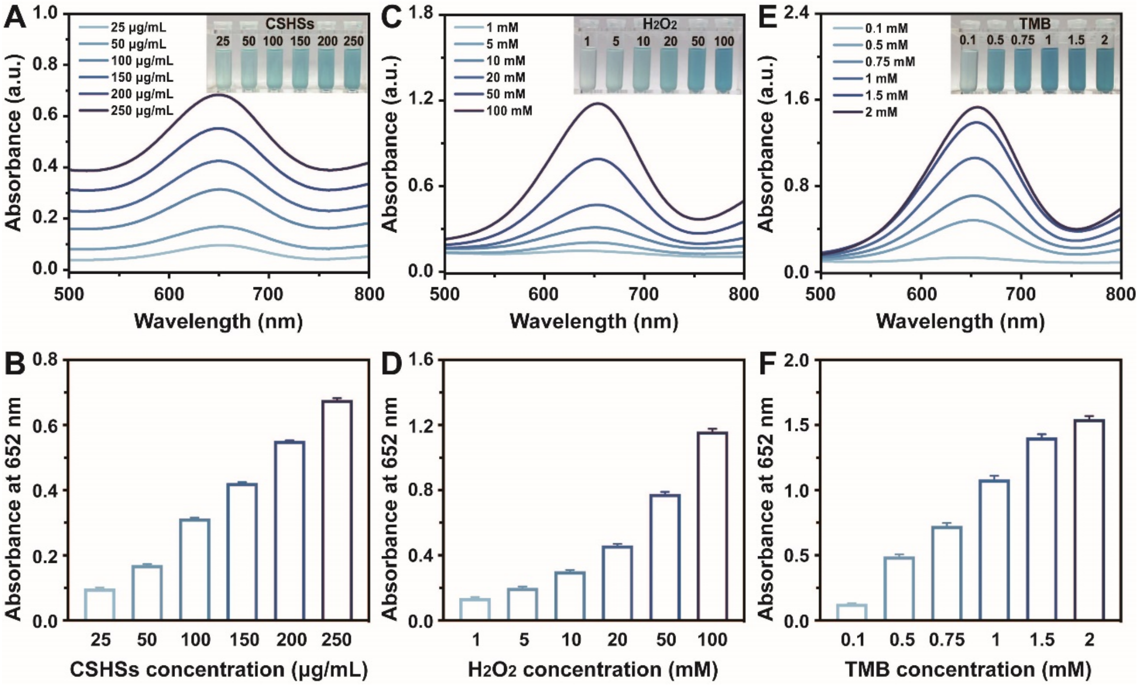

Figure S4. UV-vis absorption spectra and relative absorbance values at 652 nm of solution containing different concentrations of CSHSs (A, B), H_2O_2 (C, D), and TMB (E, F). For A and B, the concentrations of TMB and H_2O_2 were 1 mM and 10 mM. For C and D, the concentrations of CSHSs and TMB were 100 μg/mL and 1 mM. For E and F, the concentrations of CSHSs and H_2O_2 were 100 μg/mL and 100 mM. Data in (B), (D), and (F) were presented as mean ± SD (n = 3).

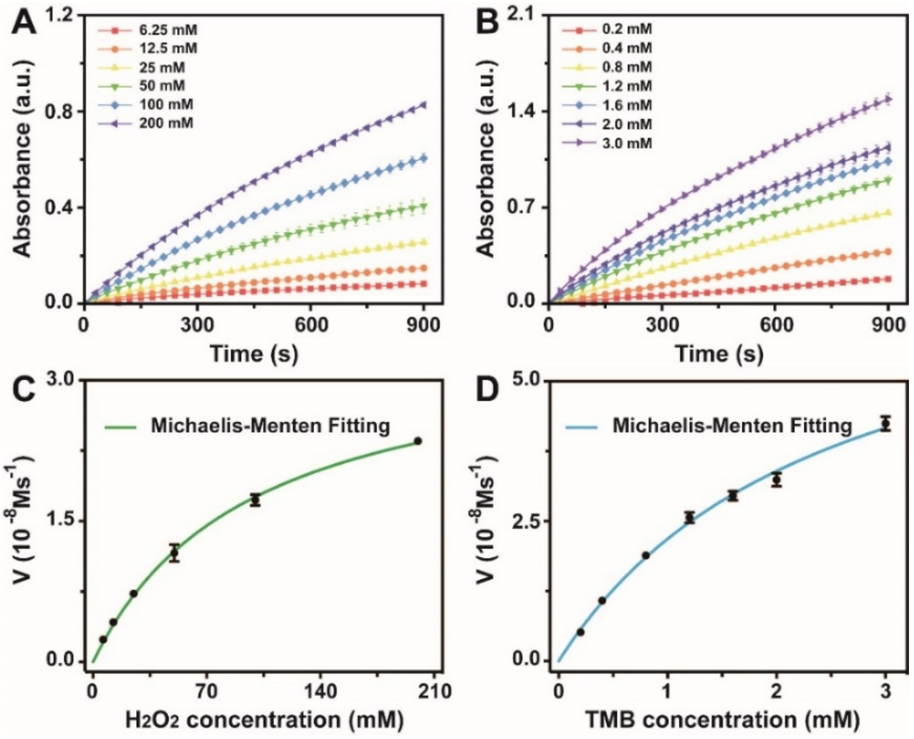

Figure S5. Time-dependent absorbance changes at 652 nm of solution containing different concentrations of H_2O_2 (A) and TMB (B). Steady-state kinetics analysis of CSHSs for H_2O_2 (C) and TMB (D). Data were presented as mean ± SD (n = 3).

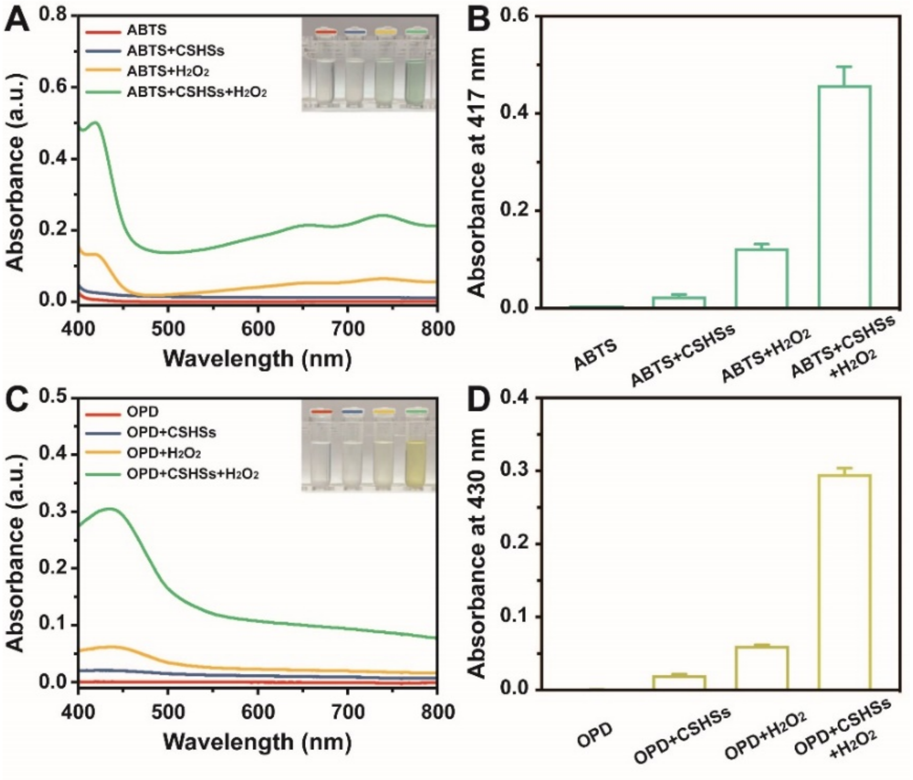

Figure S6. UV-vis absorption spectra and relative absorbance values of solutions containing ABTS (A, B) or OPD (C, D) receiving different treatments. Data in (B) and (D) were presented as mean ± SD (n = 3).

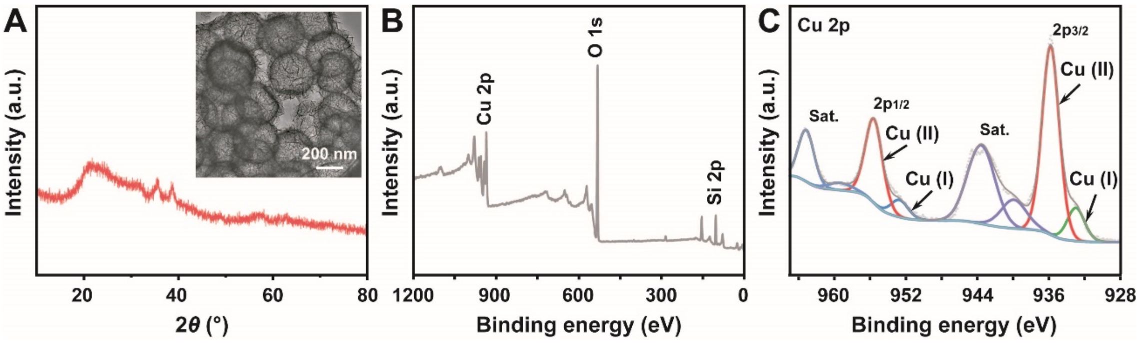

Figure S7. Wide-angel XRD pattern (A), survey XPS spectrum (B), and high-resolution Cu 2p spectrum (C) of H_2O_2-treated CSHSs. Inset of A: TEM image of CSHSs.

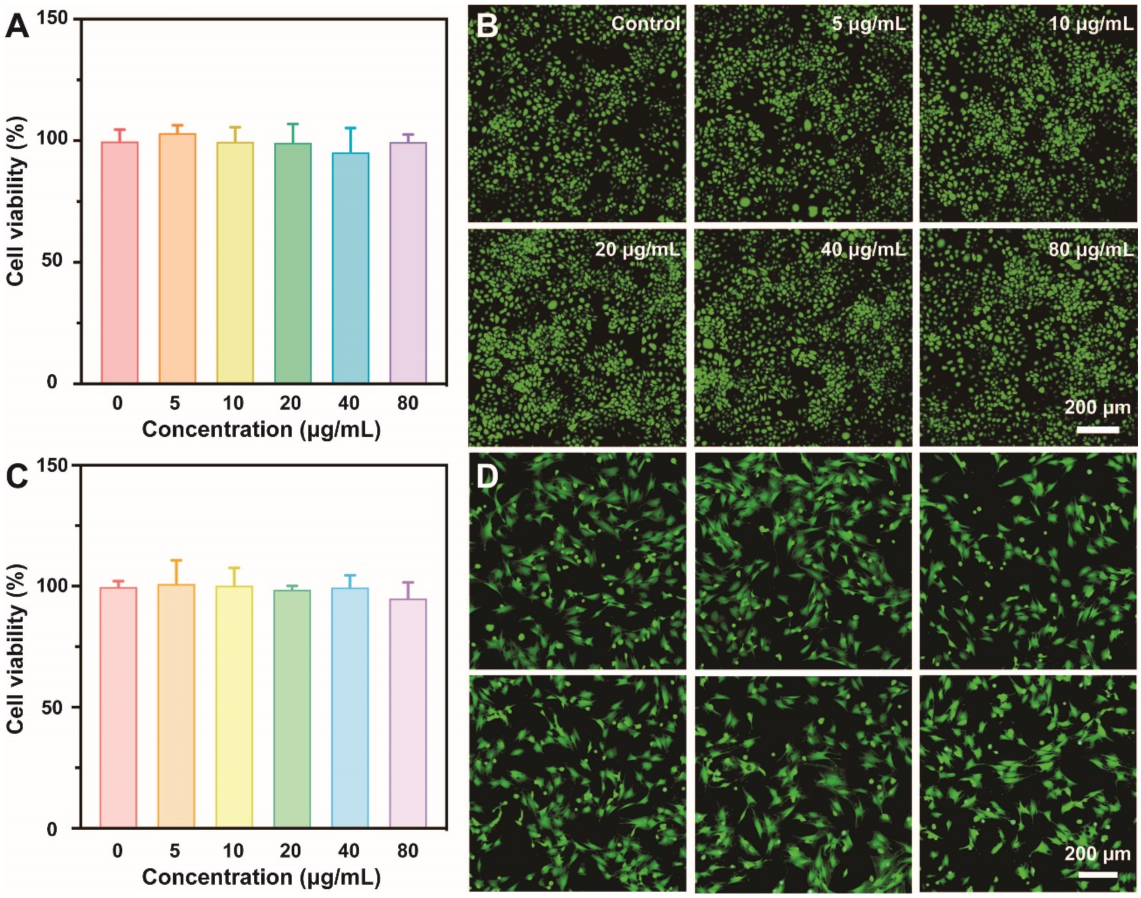

Figure S8. Viabilities of L929 cells (A) and rBMSCs (C) after being co-incubated with different concentrations of CSHSs. Live/dead staining images of L929 cells (B) and rBMSCs (D) after being co-incubated with different concentrations of CSHSs. Data in (A) and (C) were presented as mean ± SD (n = 5).

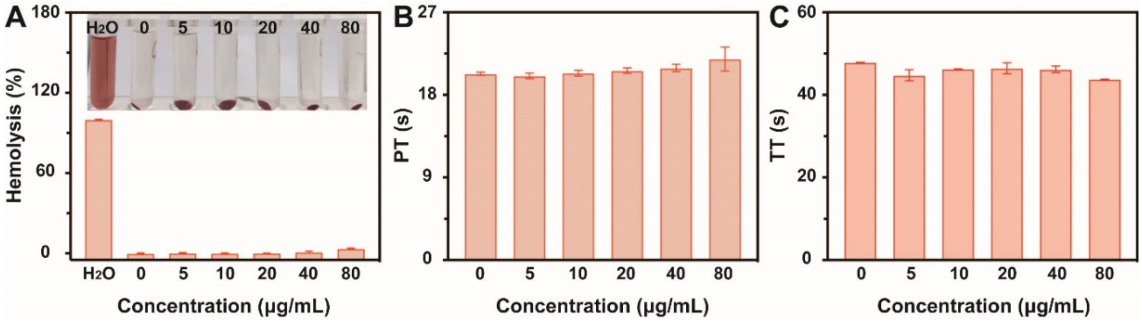

Figure S9. Concentration-dependent hemolysis (A), prothrombin time (B), and thrombin time (C) in the presence of CSHSs. Inset of A: photo of red blood cells receiving different treatments after centrifugation. Data were presented as mean ± SD (n = 3).

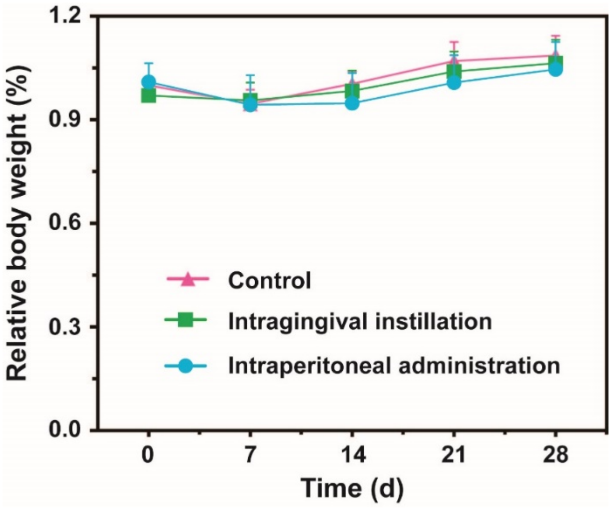

Figure S10. Time-dependent changes of mouse body weight after different administrations of CSHSs. Data were presented as mean ± SD (n = 5).

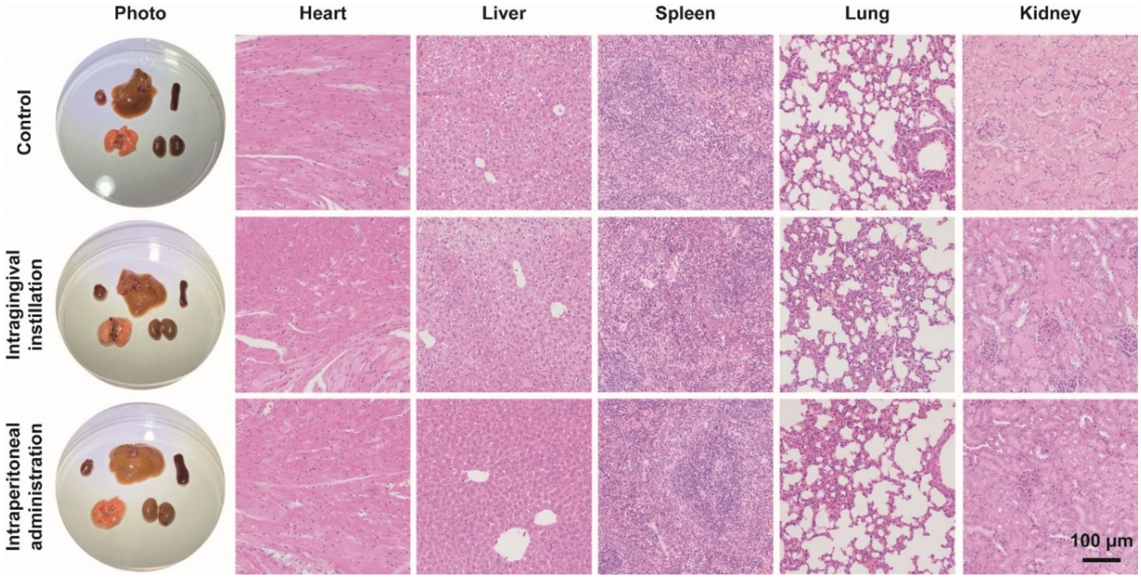

Figure S11. H&E-stained histological images and relative photos of main mouse organs after different administrations of CSHSs.

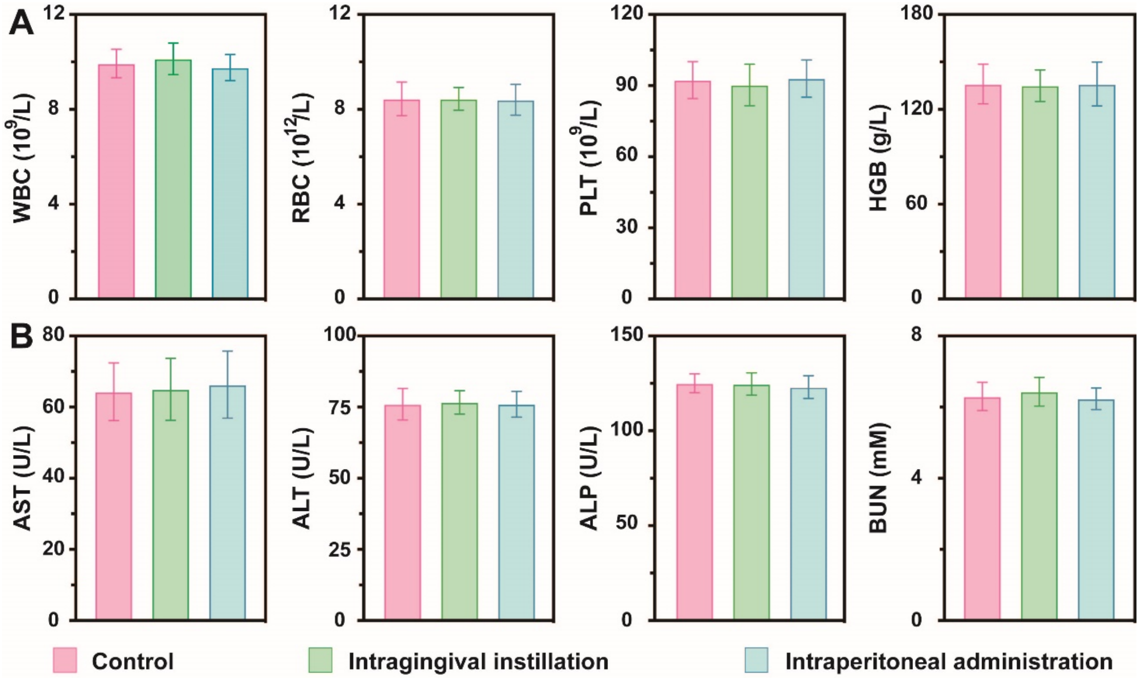

Figure S12. Changes in hematological index (A) and blood biochemical index (B) from mice after different administrations of CSHSs. WBC, white blood cell; RBC, red blood cell; PLT, platelet; HGB, hemoglobin concentration; AST, aspartate transaminase; ALT, alanine aminotransferase; ALP, alkaline phosphatase; BUN, blood urea nitrogen. Data were presented as mean ± SD (n = 3).

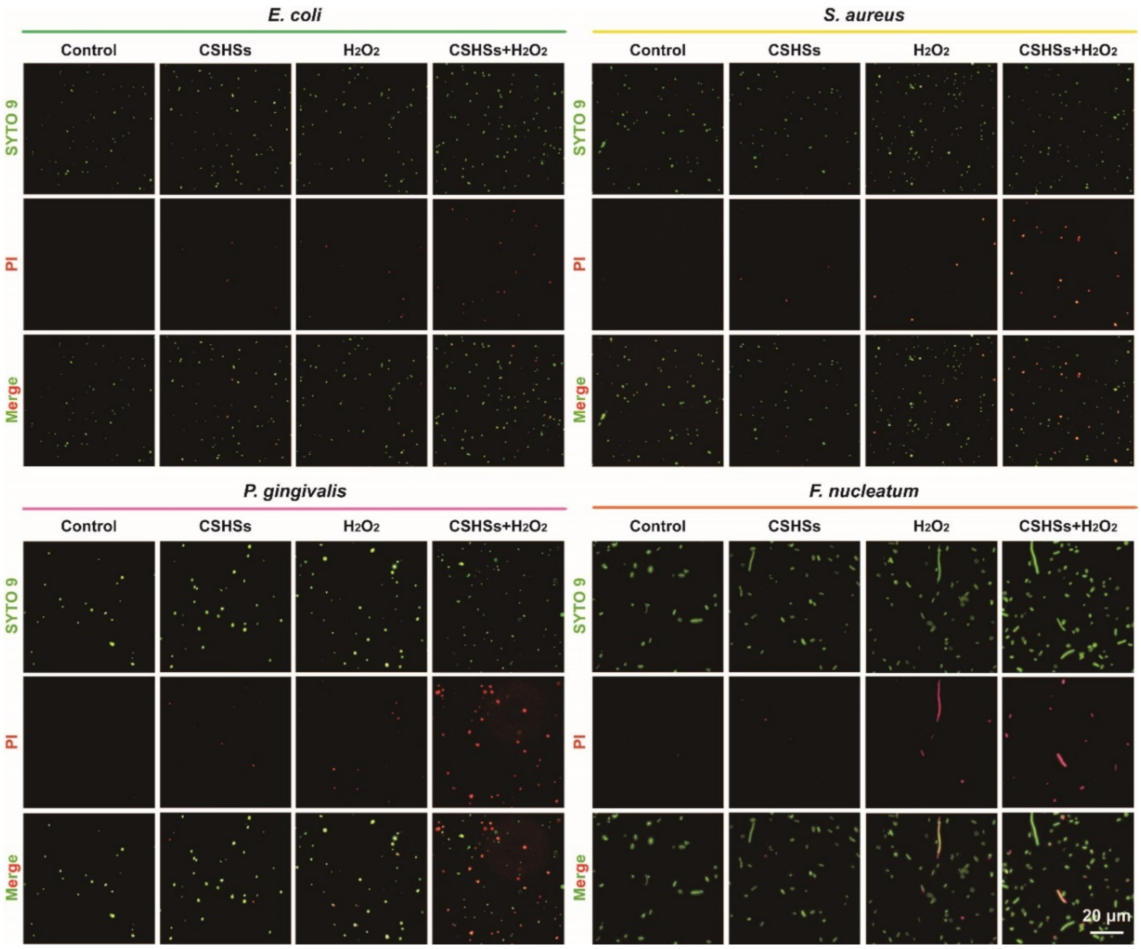

Figure S13. Live/dead staining images of various bacteria receiving different treatments.

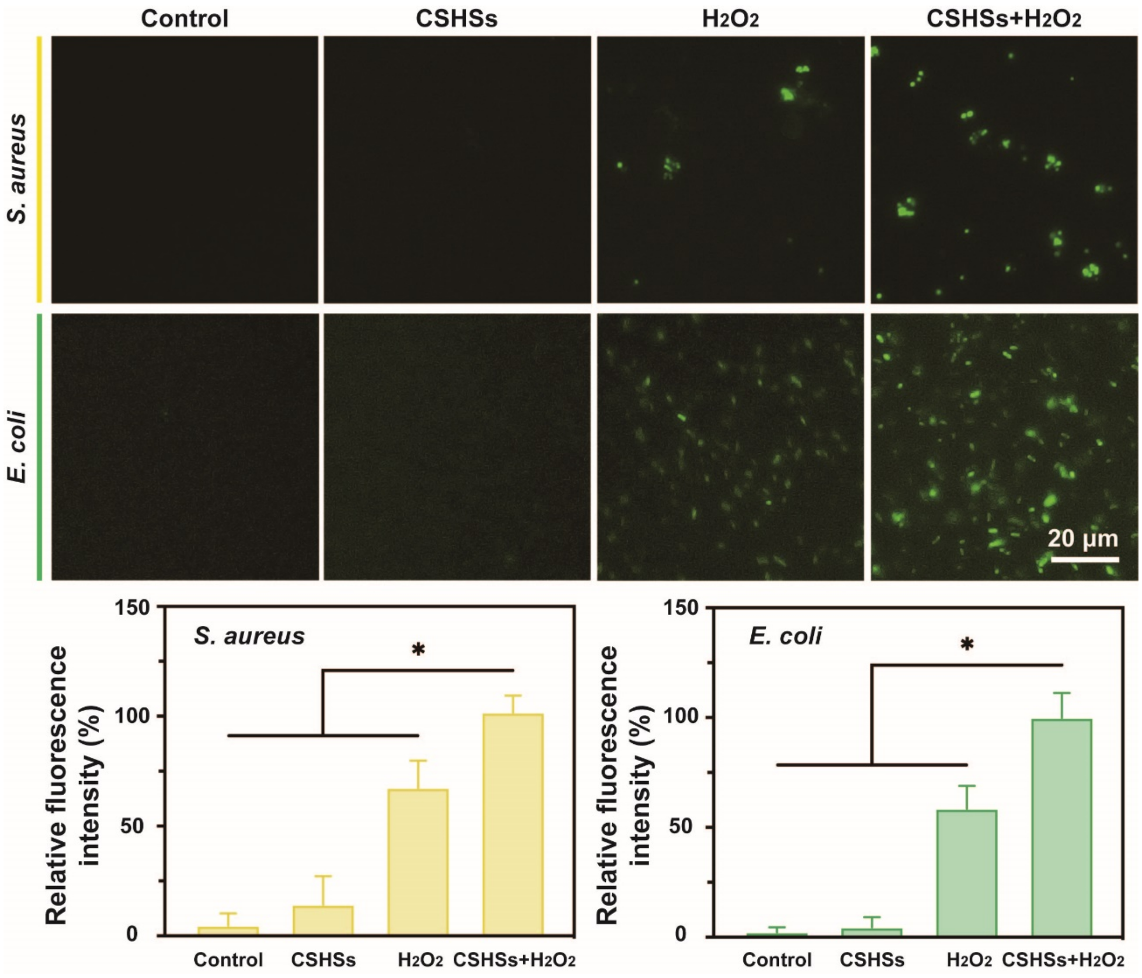

Figure S14. Intracellular ROS imaging and relative quantitative analysis in *S. aureus* and *E. coli* receiving different treatments. Data were presented as mean ± SD (n = 3). Statistical significance was calculated using one-way ANOVA with multiple comparison tests. *$p < 0.05$.

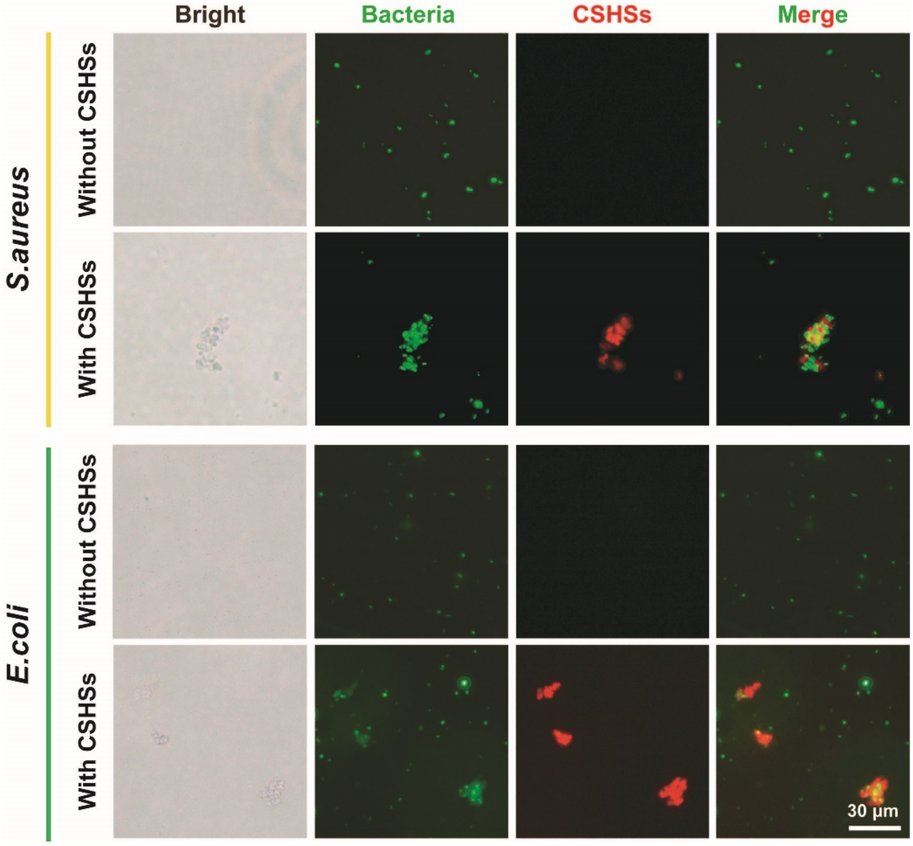

Figure S15. Fluorescence imaging for *S. aureus* and *E. coli* before and after the treatment with CSHSs. CSHSs were labelled with RB-PEG-SH while bacteria were stained with SYTO-9.

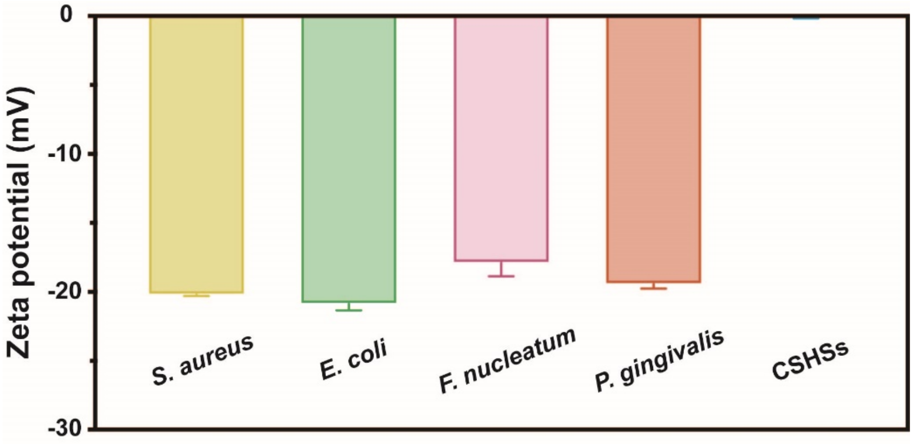

Figure S16. Zeta potentials of various bacteria and CSHSs. Data were presented as mean ± SD (n = 3).

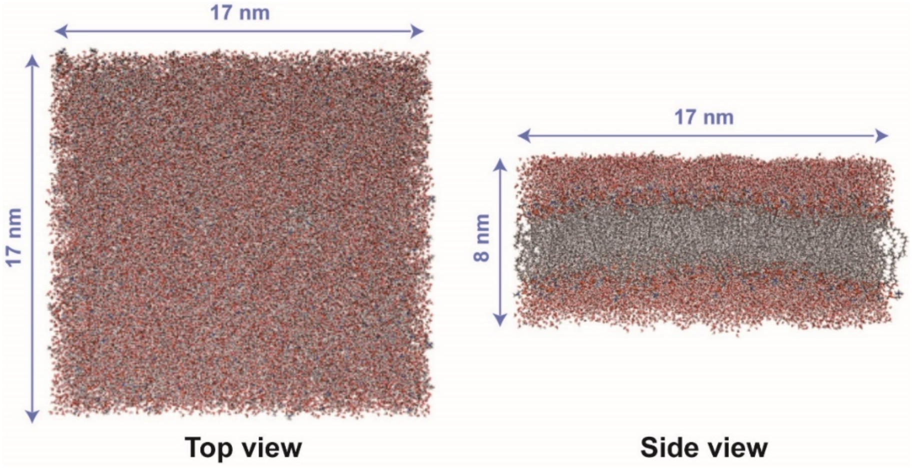

Figure S17. Top and side views of bacterial lipid membrane surrounded by H_2O.

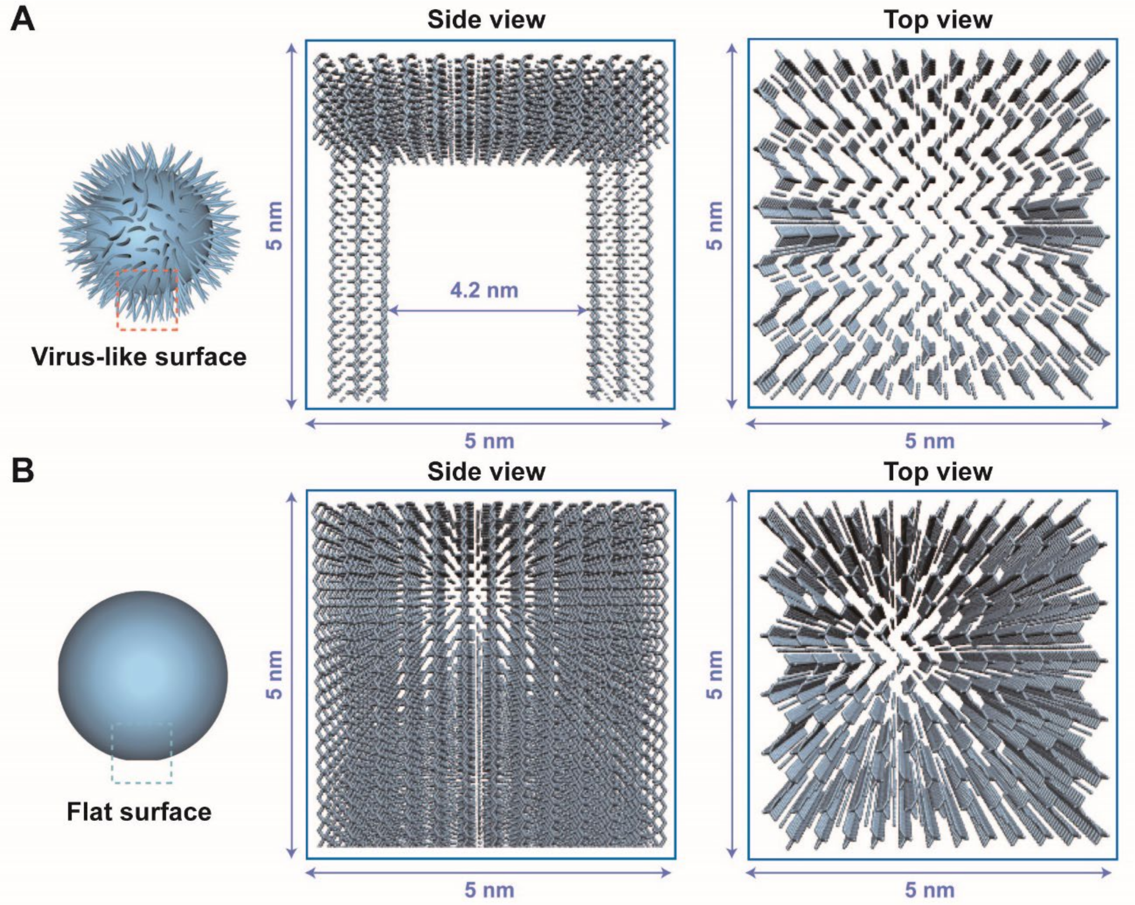

Figure S18. Scheme illustration of virus-like sphere (A) and smooth sphere (B), as well as their side and top views of the virus-like surface and flat surface.

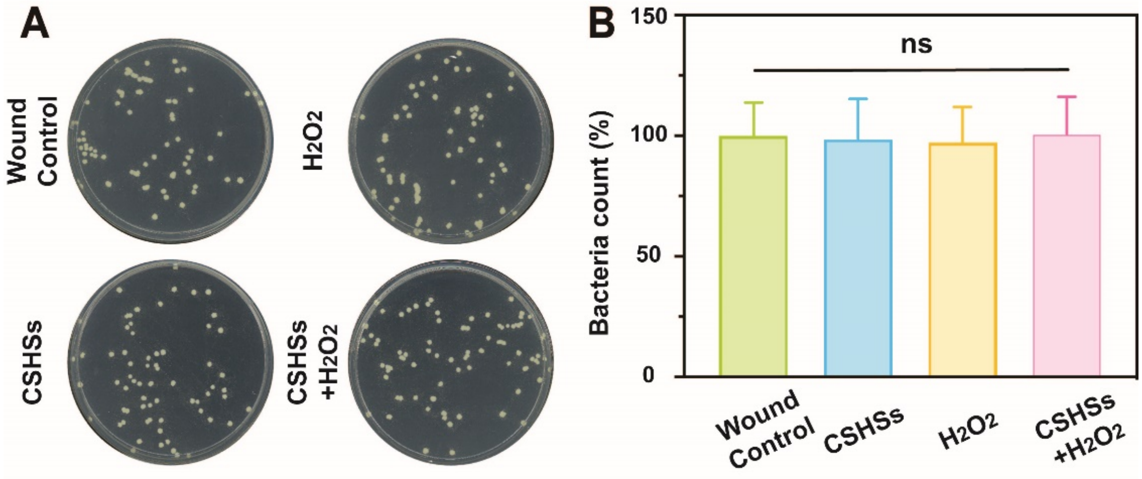

Figure S19. Photos of colonies formed by the bacteria collected from golden hamster mouths after optimal adaptive feeding and before different treatments (A) and relative bacterial numbers calculated by colony counting (B). Data in (B) were presented as mean ± SD (n = 3). Statistical significance was calculated using one-way ANOVA with multiple comparison tests. The " ns" meaned no significant difference.

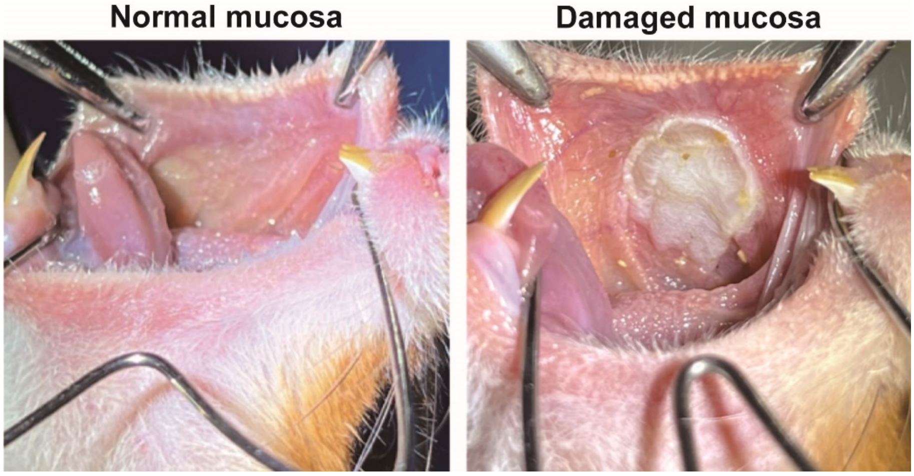

Figure S20. Photos of normal mucosa and damaged mucosa.

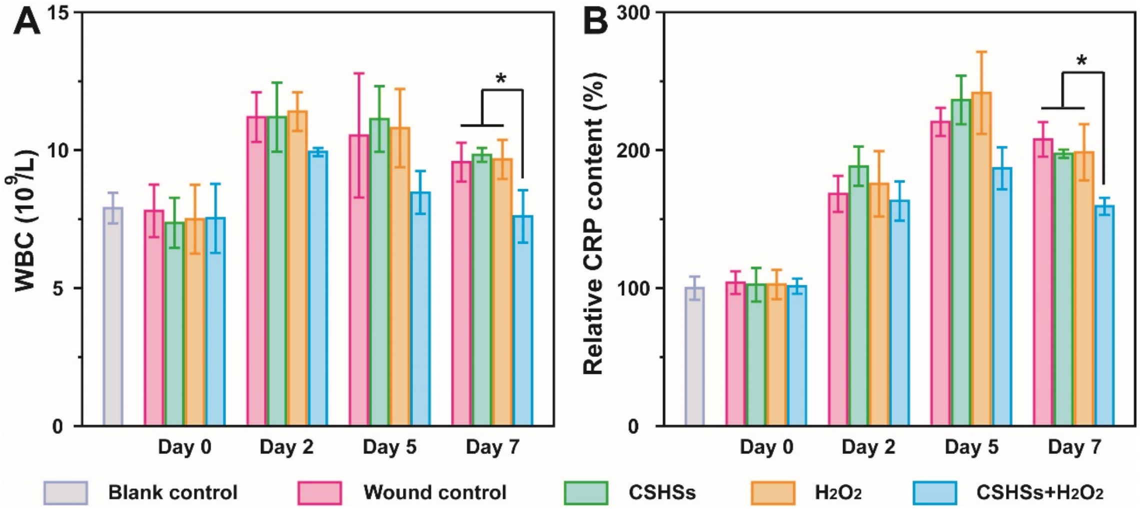

Figure S21. Time-dependent counting of white blood cells (WBCs) and relative C-reactive protein (CRP) content. Data were presented as mean ± SD (n = 3). Statistical significance was calculated using one-way ANOVA with multiple comparison tests. *$p < 0.05$.

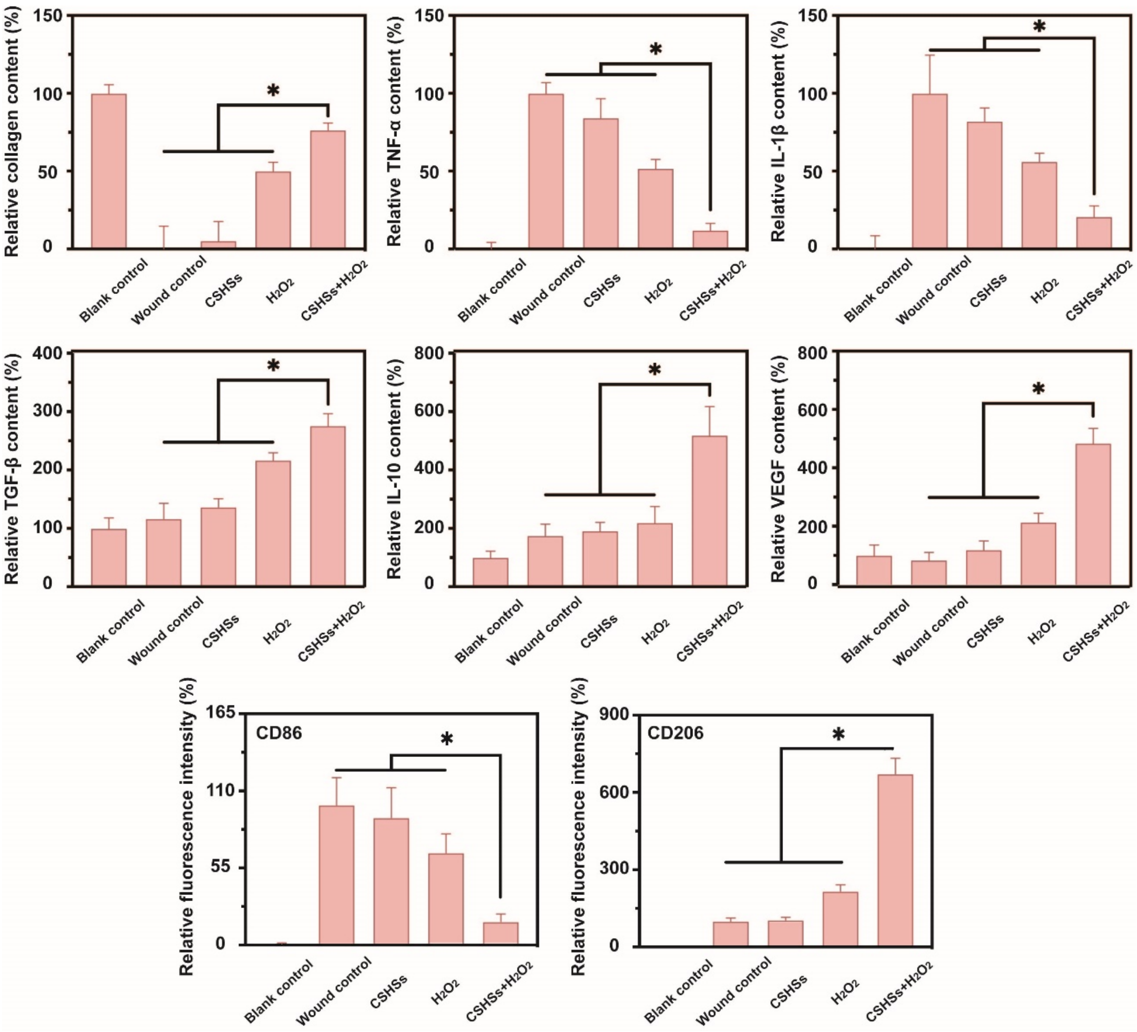

Figure S22. Quantitative analysis of collagen, TNF-α, IL-1β, TGF-β, IL-10, VEGF, CD86, and CD206 in different collected soft tissues according to Masson and immunohistochemistry/ immunofluorescence staining with the help of Image Pro Plus software. Data were presented as mean ± SD (n = 3). Statistical significance was calculated using one-way ANOVA with multiple comparison tests. *$p < 0.05$.

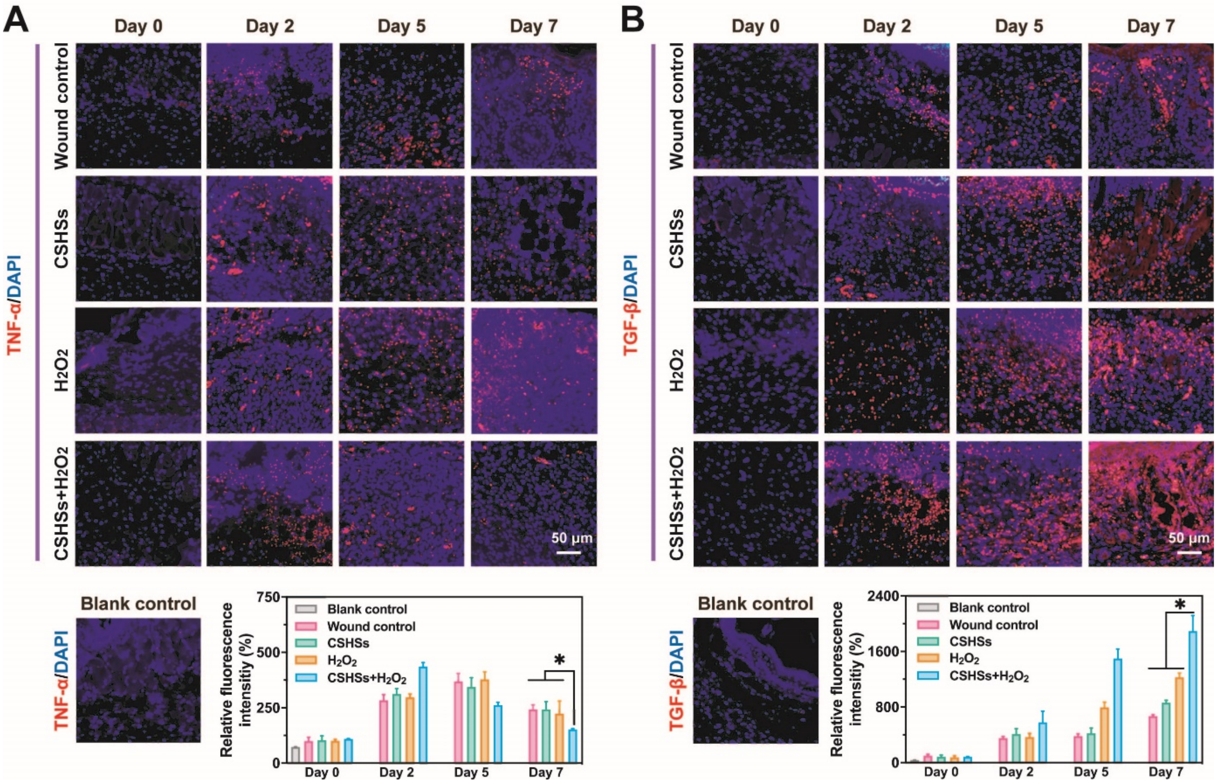

Figure S23. Time-dependent expression of TNF-α (A) and TGF-β (B) in wounds from golden hamsters with mucosal infection after different treatments and relative quantitative information. Data were presented as mean ± SD (n = 3). Statistical significance was calculated using one-way ANOVA with multiple comparison tests. **p* < 0.05.

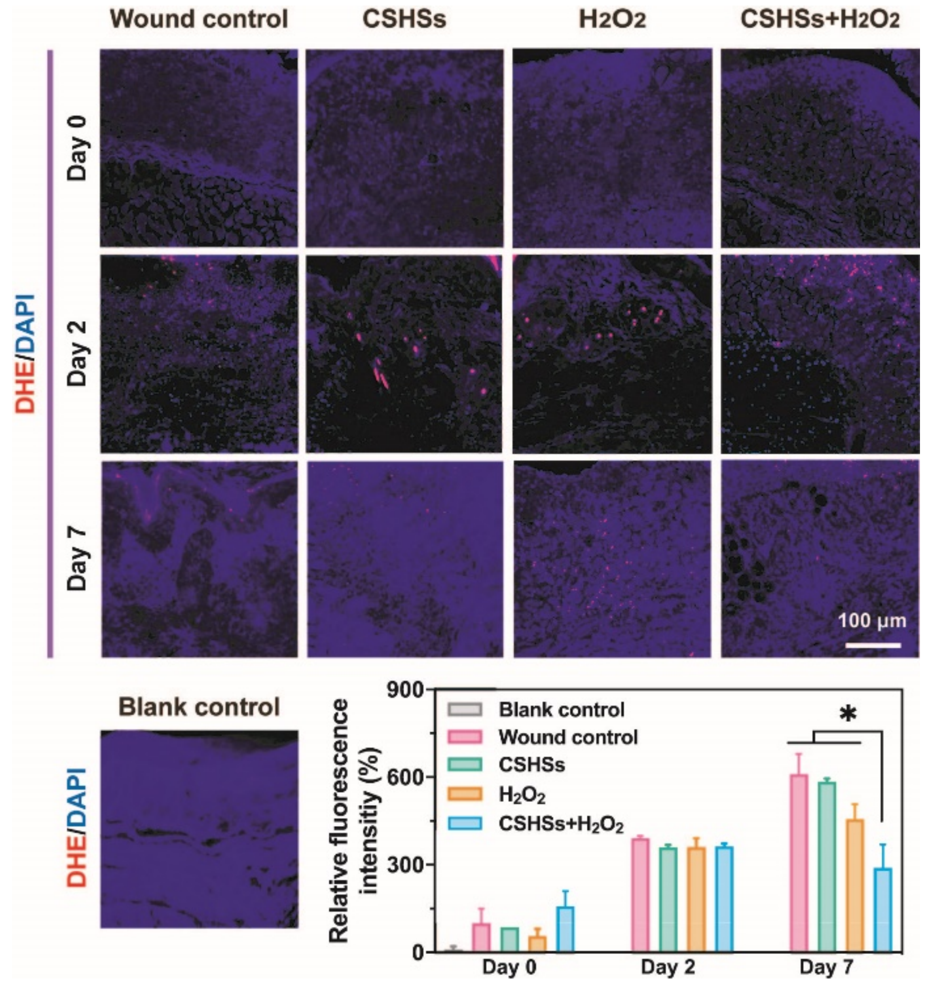

Figure S24. Time-dependent DHE staining in wounds from golden hamsters with mucosal infection after different treatments and relative fluorescence intensity. Data were presented as mean ± SD (n = 3). Statistical significance was calculated using one-way ANOVA with multiple comparison tests. $*p < 0.05$.

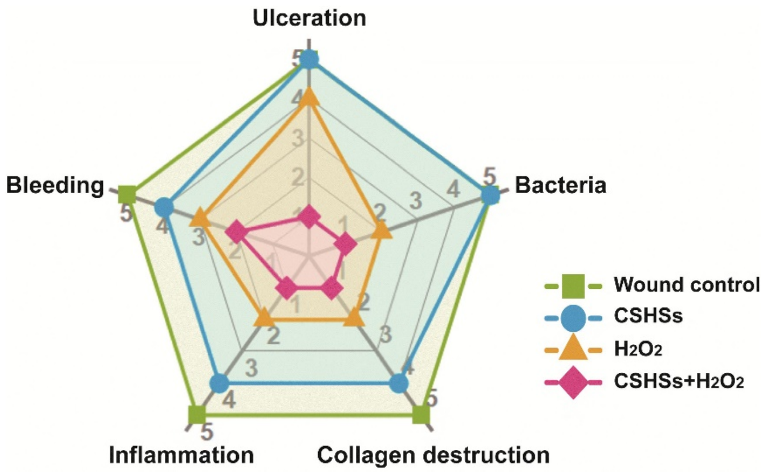

Figure S25. Mucosal damage scores for the evaluation of therapeutic effect of CSHSs+H_2O_2 system.

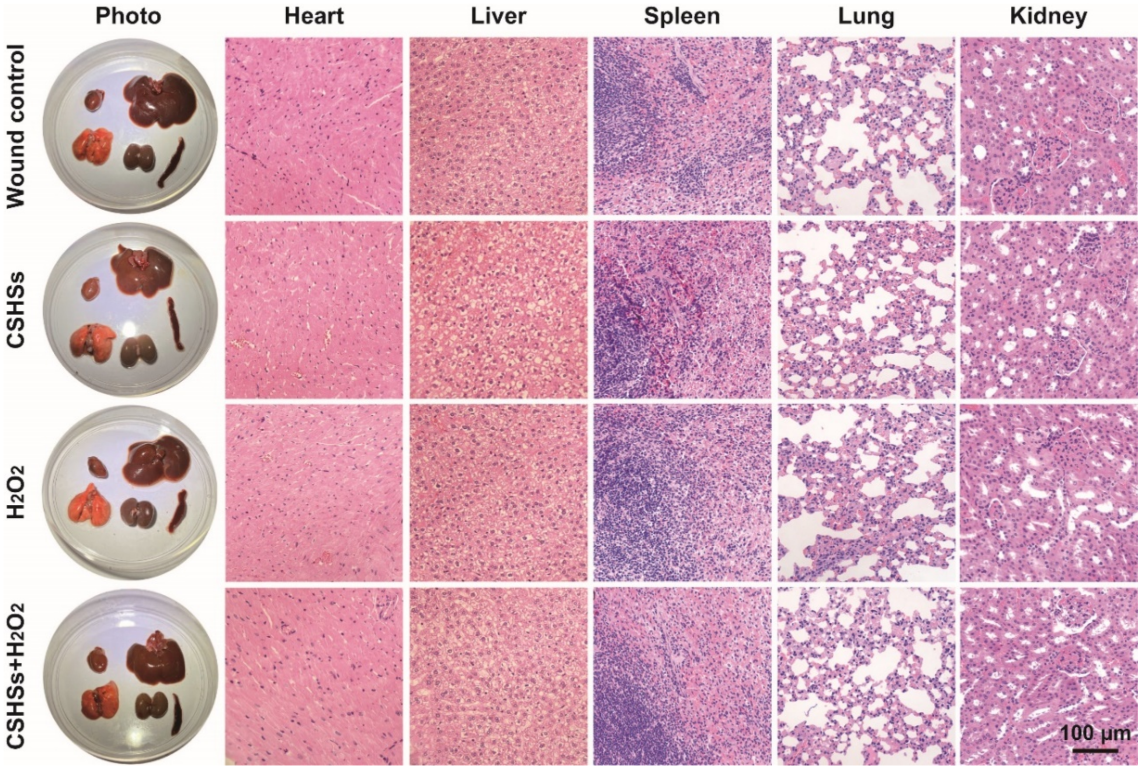

Figure S26. H&E-stained histological images and relative photos of main organs from golden hamsters with *S. aureus* infected wound after different treatments.

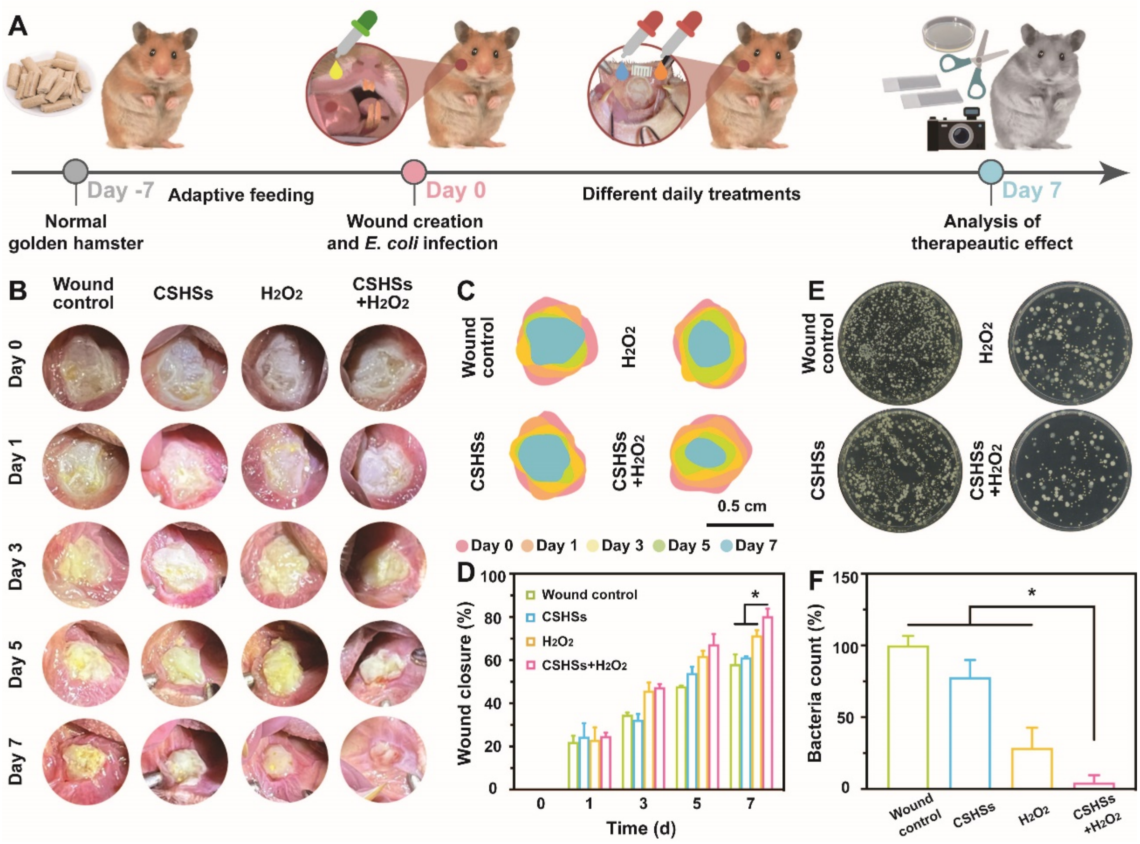

Figure S27. Schematic illustration of in vivo *E.coli*-assisted infection and different treatments in golden hamsters (A). Photos of wounds on days 0, 1, 3, 5, and 7 in golden hamsters receiving different treatments (B). Traces of wound closure over 7 days for different groups and the corresponding quantitative results (C, D). Photos of colonies formed by the bacteria collected from wounds after different treatments and the corresponding bacterial numbers calculated by colony counting (E, F). Data in (D) and (F) were presented as mean ± SD (n = 3). Statistical significance was calculated using one-way ANOVA with multiple comparison tests. $*p < 0.05$.

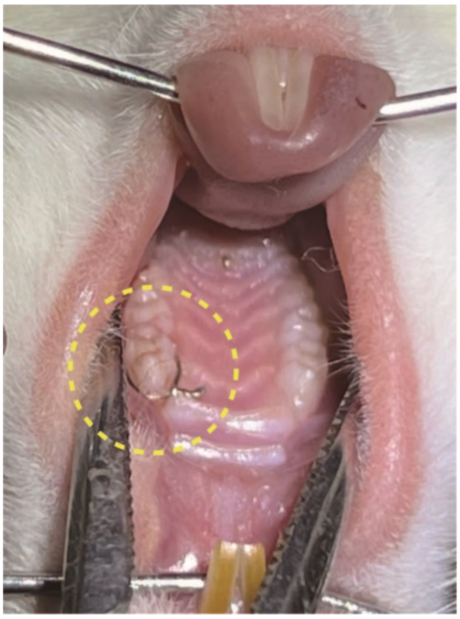

Figure S28. Photo of rat periodontitis model.

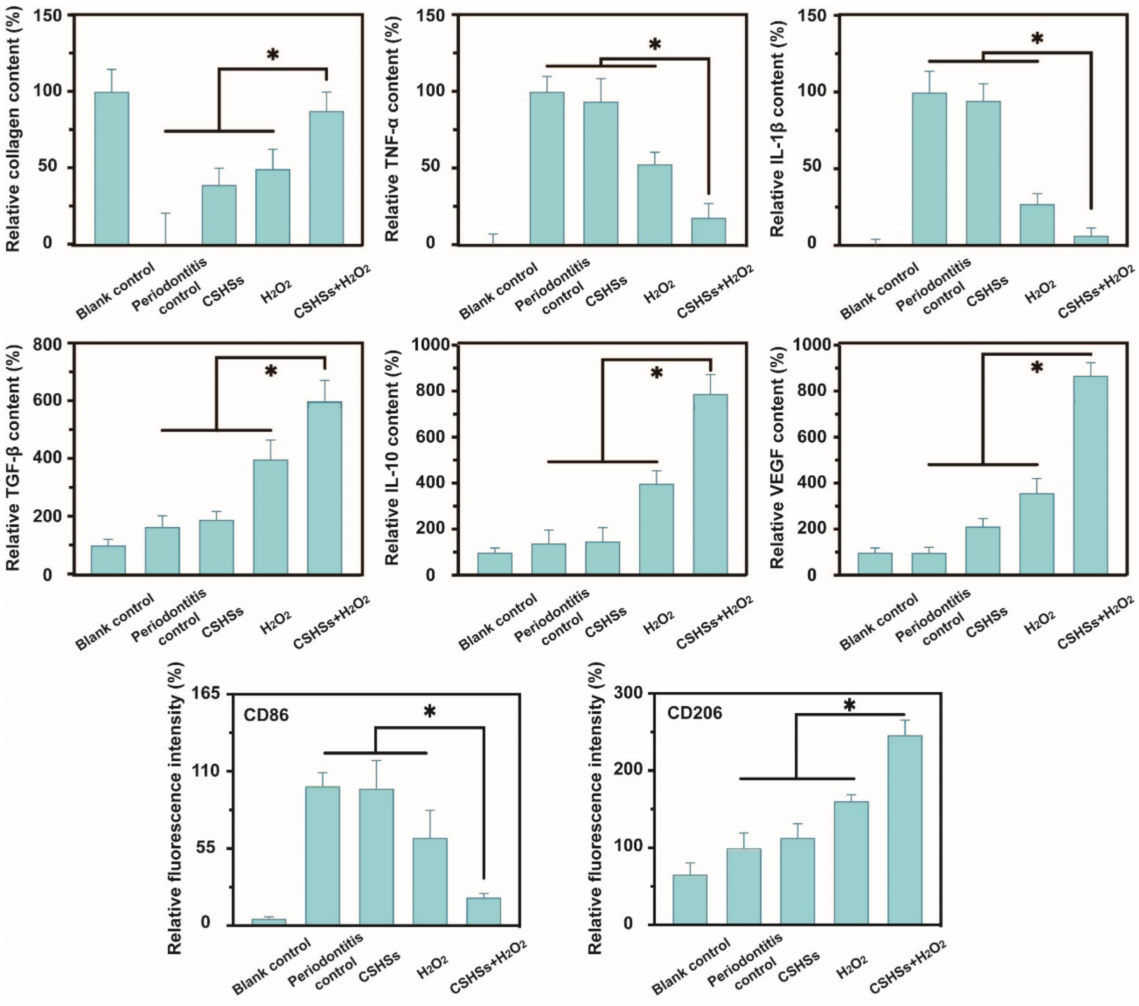

Figure S29. Quantitative analysis of collagen, TNF-α, IL-1β, TGF-β, IL-10, VEGF, CD86, and CD206 in different collected maxilla tissues according to Masson and immunohistochemistry/ immunofluorescence staining with the help of Image Pro Plus software. Data were presented as mean ± SD (n = 3). Statistical significance was calculated using one-way ANOVA with multiple comparison tests. *$p < 0.05$.

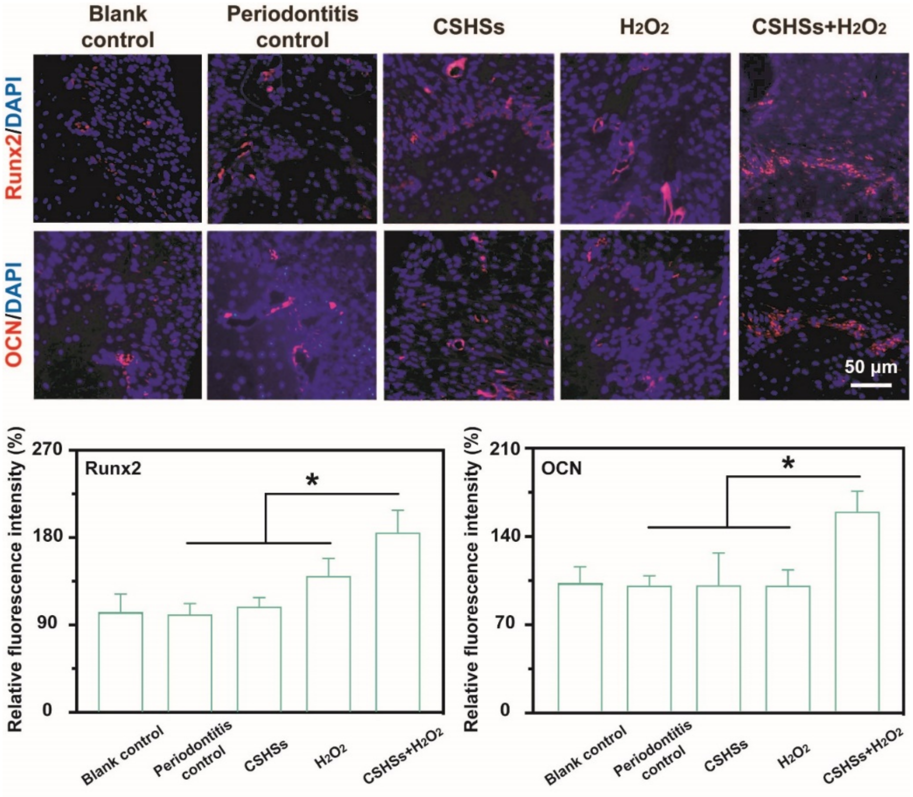

Figure S30. Immunofluorescence staining and relative quantitative analysis of Runx2 and OCN in different collected tissues from rats with periodontitis after different treatments. Data were presented as mean ± SD (n = 3). Statistical significance was calculated using one-way ANOVA with multiple comparison tests. *$p < 0.05$.

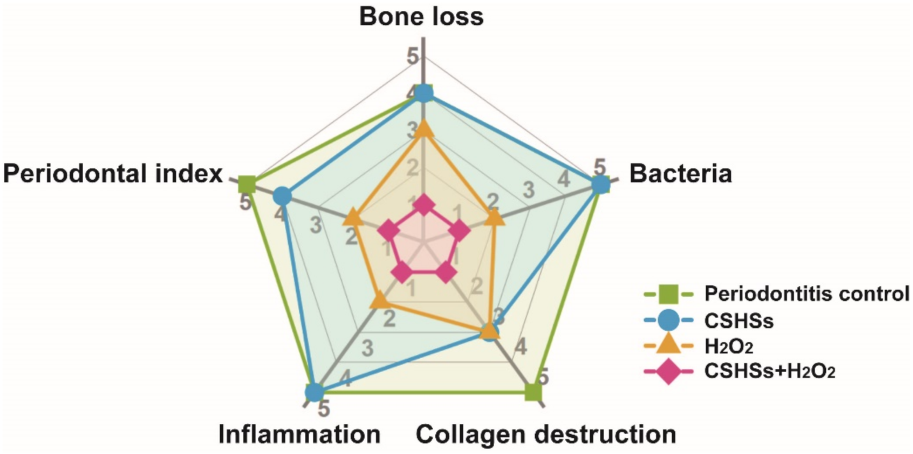

Figure S31. Periodontal damage scores for the evaluation of therapeutic effect of CSHSs+H_2O_2 system.

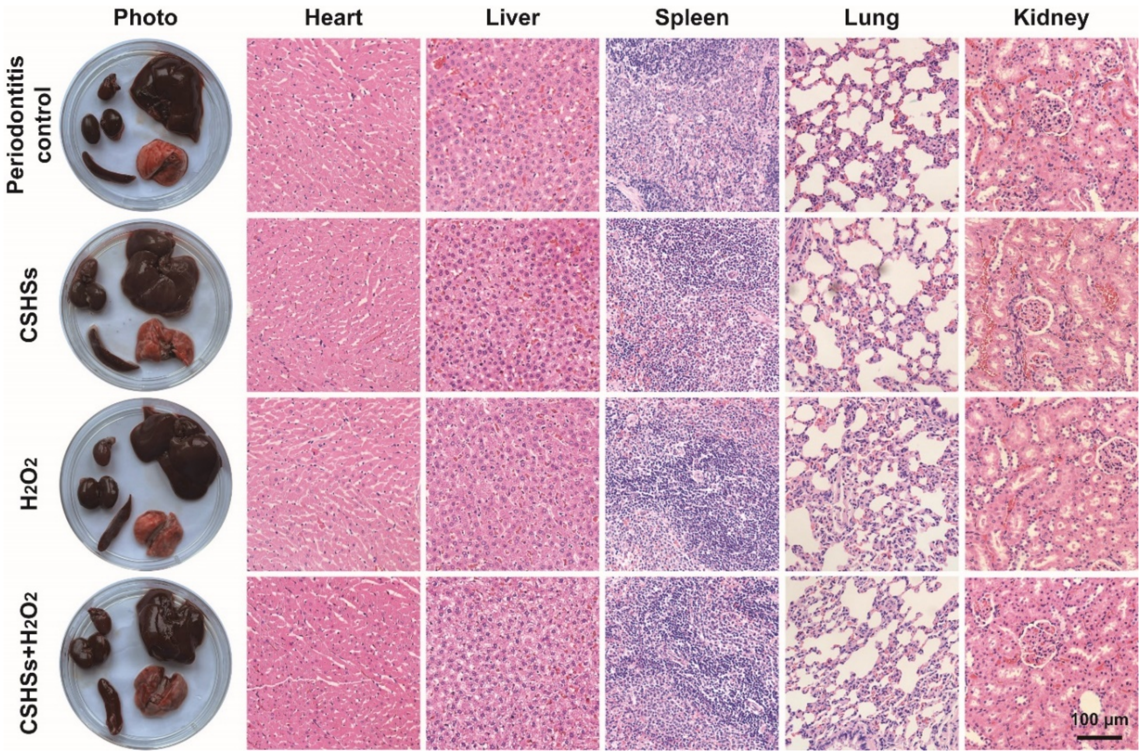

Figure S32. H&E-stained histological images and relative photos of main organs from rats with periodontitis after different treatments.

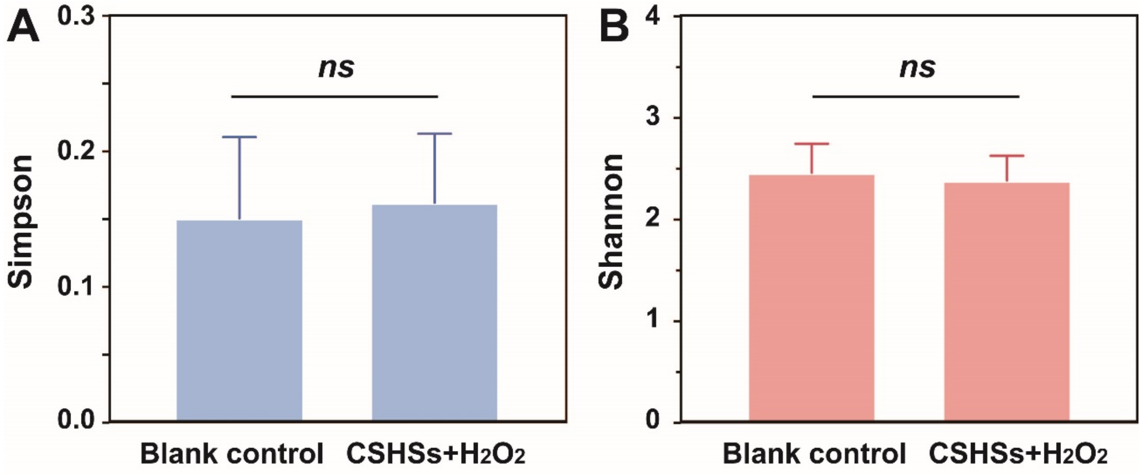

Figure S33. The α-diversity of oral microbiome illustrated by Simpson's index (A) and Shannon's index (B). Data were presented as mean ± SD (n = 3). Statistical significance was calculated using unpaired Student's *t*-test. *ns*, not significant.

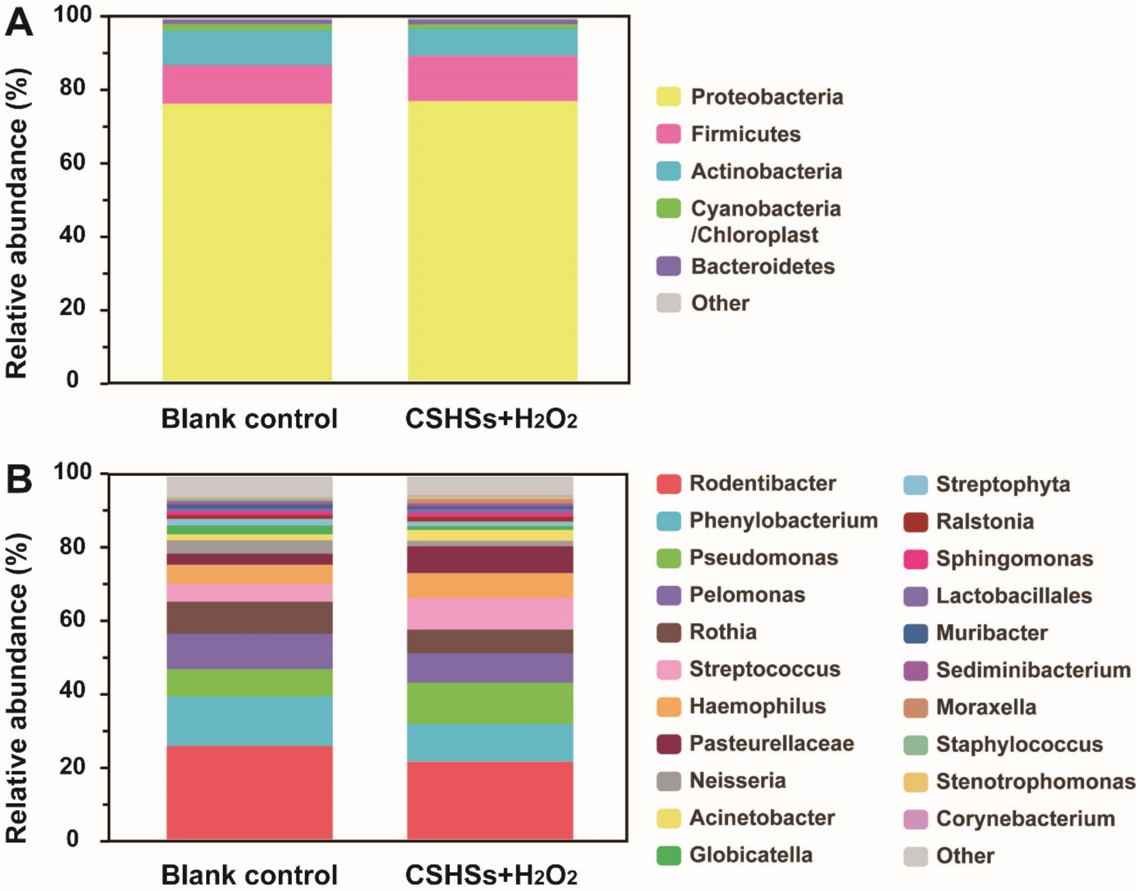

Figure S34. Distribution of oral microbial communities of different groups at the phylum level (A) or the genus level (B).

Table S1. Summary of the kinetic parameters of CSHSs and HRP.

Catalysts	Substrates	K_m (mM)	V_{max} (M/s)
CSHSs	H_2O_2	97.31	3.47×10^{-8}
	TMB	2.492	7.625×10^{-8}
HRP	H_2O_2	3.7	8.71×10^{-8}
	TMB	0.434	10.00×10^{-8}

Table S2. Average hemolysis rates 3 h after incubation with CSHSs.

Concentration (μg/mL)	Hemolysis (%)
0.9% NaCl	0
H2O	100
5	0.30
10	0.10
20	0.18
40	1.01
80	3.61

Table S3. Average blood coagulation results after incubation with CSHSs.

Concentration (μg/mL)	PT (s)	TT (s)
0.9% NaCl	20.30	47.87
5	20.07	44.73
10	20.37	46.27
20	20.63	46.20
40	20.93	46.43
80	21.90	43.77

Table S4. Urine test results of mice in the groups of control, intragingival instillation group and intraperitoneal administration group.

Group	WBC (cells/μL)	protein (g/L)	pH value	blood (cells/μL)	specific gravity	bilirubin (μmol/L)
Control	-	-	6.0	-	1.03	-
Intragingival instillation	-	-	6.0	-	1.03	-
Intraperitoneal administration	-	-	6.0	-	1.03	-

Table S5. Criteria of radar chart scoring parameters in oral mucosal wound model.

Scores	0	1	2	3	4	5
Ulceration (Relative wound area)	No ulceration (0%)	Superficial ulcerations (0%~12%)	Mild ulcerations (12%~15%)	Moderate ulcerations (15%~18%)	Considerable ulcerations (18%~21%)	Deep ulcerations (>21%)
Bacteria (Relative bacteria number)	Absence of bacteria (~15%)	Minimal number of bacteria (15%~30%)	Minimal/medium number of bacteria (30%~45%)	Medium number of bacteria (45%~60%)	Medium number of/plentiful bacteria (60%~75%)	Plentiful bacteria (>75%)
Collagen destruction	Plentiful collagen deposition (>85%)	Medium/plentiful collagen deposition (65%~85%)	Medium collagen deposition (45%~65%)	Minimal/medium collagen deposition (25%~45%)	Minimal collagen deposition (5%~25%)	Absence of collagen deposition (<5%)
Inflammation (Relative TNF-α positive expression)	Absence of inflammation (<10%)	Minimal inflammatory cell infiltration (10%~30%)	Mild inflammatory cell infiltration (30%~50%)	Moderate inflammatory cell infiltration (50%~70%)	Marked inflammatory cell infiltration (70%~90%)	Severe inflammatory cell infiltration (>90%)
Bleeding	Healthy	No bleeding	Slight bleeding	Mild bleeding	Moderate bleeding	Severe bleeding

Table S6. Criteria of radar chart scoring parameters in experimental periodontitis model.

Scores	0	1	2	3	4	5
Bone loss (CEJ-ABC)	Normal (<0.50 mm)	Bland bone loss (0.50~0.55 mm)	Mild/moderate bone loss (0.55~0.60 mm)	Moderate bone loss (0.60~0.65 mm)	Moderate/severe bone loss (0.65~0.70 mm)	Severe bone loss (>0.70 mm)
Bacteria (Relative bacteria number)	Absence of bacteria (<10%)	Minimal number of bacteria (10%~30%)	Minimal/medium number of bacteria (30%~50%)	Medium number of bacteria (50%~70%)	Medium number of/plentiful bacteria (70%~90%)	Plentiful bacteria (>90%)
Collagen destruction	Plentiful collagen deposition (>90%)	Medium/plentiful collagen deposition (70%~90%)	Medium collagen deposition (50%~70%)	Minimal/medium collagen deposition (30%~50%)	Minimal collagen deposition (10%~30%)	Absence of collagen deposition (<10%)
Inflammation (Relative IL-1β positive expression)	Absence of inflammation (<5%)	Minimal inflammatory cell infiltration (5%~25%)	Mild inflammatory cell infiltration (25%~45%)	Moderate inflammatory cell infiltration (45%~65%)	Marked inflammatory cell infiltration (65%~85%)	Severe inflammatory cell infiltration (>85%)
Periodontal condition	Healthy	No gingival bleeding	Gingival bleeding on probing	Spontaneous gingival bleeding	Shallow periodontal pocket	Deep periodontal pocket
